# Long-distance endosome trafficking drives fungal effector production during plant infection

**DOI:** 10.1038/ncomms6097

**Published:** 2014-10-06

**Authors:** Ewa Bielska, Yujiro Higuchi, Martin Schuster, Natascha Steinberg, Sreedhar Kilaru, Nicholas J. Talbot, Gero Steinberg

**Affiliations:** 1 School of Biosciences, College of Life and Environmental Sciences, University of Exeter, Stocker Road, Exeter EX4 4QD, UK

## Abstract

To cause plant disease, pathogenic fungi can secrete effector proteins into plant cells to suppress plant immunity and facilitate fungal infection. Most fungal pathogens infect plants using very long strand-like cells, called hyphae, that secrete effectors from their tips into host tissue. How fungi undergo long-distance cell signalling to regulate effector production during infection is not known. Here we show that long-distance retrograde motility of early endosomes (EEs) is necessary to trigger transcription of effector-encoding genes during plant infection by the pathogenic fungus *Ustilago maydi*s. We demonstrate that motor-dependent retrograde EE motility is necessary for regulation of effector production and secretion during host cell invasion. We further show that retrograde signalling involves the mitogen-activated kinase Crk1 that travels on EEs and participates in control of effector production. Fungal pathogens therefore undergo long-range signalling to orchestrate host invasion.

## Abstract (web summary)

It is unclear how the nuclei of very long fungal cells (hyphae) receive information from the hyphal tips during the invasion of plant tissues. Here, the authors show that retrograde movement of early endosomes, from the hyphal tip to the nucleus, is required for this signalling process.

## Graphical abstract

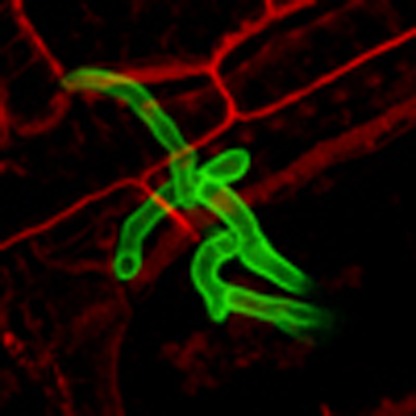

Plant pathogenic fungi invade their host plants by hyphal tip growth, which is an important determinant of fungal virulence. In response to infection, plant innate defences recognize pathogens and trigger a complex set of physiological responses^1^,^2^. Fungal pathogens overcome host immunity by secretion of a battery of effector proteins^3^,^4^,^5^. These effector proteins suppress plant immunity, thereby allowing rapid fungal colonization of plant tissue. Such interplay between pathogen and host forms the basis of the biotrophic interaction of the corn smut fungus *Ustilago maydis* and maize^6^. In this interaction, the fungus explores the plant surface by tip growth, but upon recognition of host surface cues initiates penetration of plant epidermal cells^6^,^7^. During this initial phase of development, a number of plant defence genes are induced, suggesting that the host recognizes the fungal intruder^8^. However, the host defence reaction is suppressed rapidly by secreted fungal effectors. A subset of these have been studied in depth, such as Pep1, required for penetration and for inhibition of apoplastic plant peroxidases^9^,^10^, Pit2, which inhibits apoplastic plant proteases^11^,^12^ and Cmu1, a chorismate mutase, which attenuates plant salicylic acid levels^13^ and thus suppresses plant defence responses.

As plant defence responses occur within minutes of perception of a pathogen^14^,^15^,^16^, fungal effector proteins must be produced and secreted equally rapidly. Effector production must therefore be induced as soon as the hyphal tip begins invasion. However, the architecture of an invading hyphal cell poses a challenge, because the nucleus is located at a considerable distance from the invading tip^17^. It must therefore perceive a long-range signal from the plasma membrane to induce effector transcription. In animal neurons, such retrograde signalling from the synapse to the nucleus is mediated by early endosomes (EEs). They deliver signalling components, including mitogen-activated protein kinases (MAPKs; ref. 18) along microtubules to the nucleus^19^,^20^,^21^. Microtubule-dependent EE motility was described in *U. maydis*^22^,^23^ and other fungi^24^,^25^. Individual organelles can travel up to 90 μm, and therefore potentially traverse the distance between the hyphal tip and nucleus^26^,^27^. However, a role for fungal EEs in cell signalling has not been reported.

Here, we focus on the mechanism of long-range signalling during early infection of maize by *U. maydis*. We present evidence that effectors are secreted at the invading hyphal tip. We have developed a method to immobilize EEs very specifically, and we show that EE motility is crucial for transcription and subsequent secretion of the Cmu1, Pep1 and Pit2 effectors. EEs therefore transmit signals to the nucleus to regulate plant infection. Moreover, we provide evidence that the MAPK Crk1 localizes to moving EEs and that effector production and secretion is deregulated when *crk1* is deleted. Collectively, our results are consistent with a role for motor-driven EE motility in retrograde signalling during infection. This long-range signalling mechanism is therefore of pivotal importance to fungal pathogenicity.

## Results

### Fungal EEs move retrogradely during plant infection

**Figure 1:**
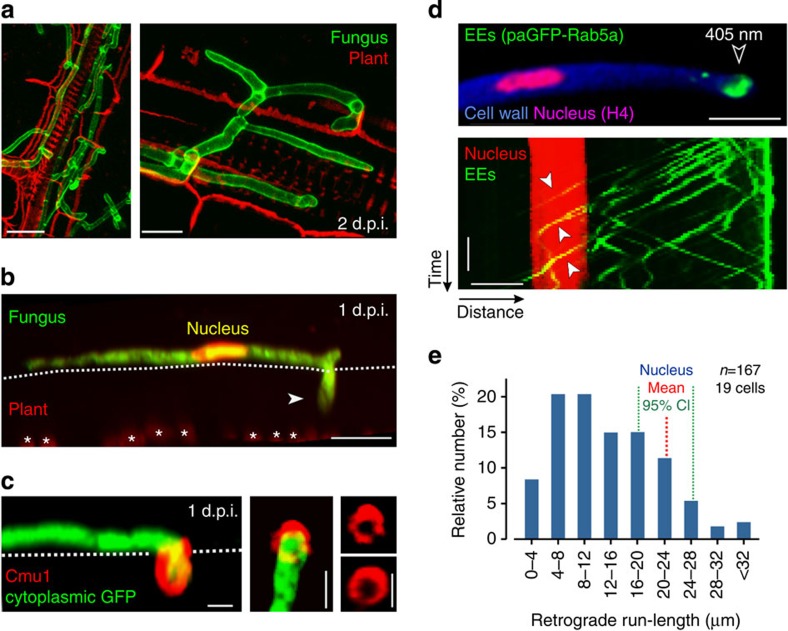
Endosomes travel from the invading hyphal tip to the nucleus. (**a**) Lectin-stained hyphal cells (green) colonizing a leaf at 2 d.p.i. Plant cell walls were stained using propidium iodide (red). Scale bars, 20 μm (left panel) and 10 μm (right panel). (**b**) Invasion of hyphal cells, expressing a nuclear RFP (nucleus; appearing yellow) and cytoplasmic GFP (green), at 1 d.p.i. Dotted line indicates plant surface. Chloroplasts visualized by their auto-fluorescence (asterisks, red). Arrow head points towards invading hyphal tip. Scale bar, 10 μm. See Supplementary Movie 1. (**c**) Secretion of effector protein Cmu1-mCherry (red) during early fungal invasion (fungus in green). Right panels reveal Cmu1-mCherry at the fungus–plant interface. Scale bars, 2 μm. See Supplementary Movie 2. (**d**) Image and kymograph show retrograde motility of photo-activated paGFP-Rab5a-labelled EEs (green) towards sub-apical nucleus (red, labelled with histone4-mCherry) in an invading fungal cell. The fungal cell wall was stained with Calcofluor White (blue). Point of photo-activation indicated by open arrowhead. Solid arrows in lower panel indicate long range motility. Scale bars, 10 μm (upper panel), 3 s (vertical, lower panel) and 5 μm (horizontal, lower panel). (**e**) Retrograde run-length of photo-activated EEs in invading hyphae (1 d.p.i.). Mean position of nucleus is indicated in red; 95% confidence intervals (CIs) indicated by green dotted line. Sample size from >3 experiments is shown.

The corn smut fungus, *U. maydis* colonizes plant tissues using invasive hyphae (Fig. 1a) that grow at their tips. During infection, the fungus secretes a battery of effector proteins^9^,^11^,^12^,^13^,^28^,^29^. These effector proteins allow the pathogen to remain masked from plant recognition, thereby overcoming plant defence. We visualized the fungal nucleus, during the initial phase of plant infection, by expressing triple red fluorescent protein (RFP) fused to a nuclear localization signal in cells that also expressed cytoplasmic green fluorescent protein (GFP; for all genotypes see Table 1; and for usage of strains see Supplementary Table 1). We found the nucleus routinely located 22.00±2.01 μm behind the distal invading tip (Fig. 1b and Supplementary Movie 1). To investigate effector secretion, we visualized the metabolic effector chorismate mutase Cmu1 (ref. 13). We tested whether Cmu1 is secreted upon host penetration by fusing mCherry to the endogenous *cmu1* gene and expressing the translational fusion in a *U. maydis* strain that co-expressed cytoplasmic GFP. This did not impair virulence, demonstrating that the fusion protein is fully functional (Supplementary Fig. 1a,b). Cmu1 surrounded the invading hyphal tip (Fig. 1c), consistent with its role in suppressing plant defence^13^. Significantly, this occurred at a long distance from the nucleus of the invading hypha that remained on the leaf surface (Fig. 1b and Supplementary Movie 1). We then tested whether EE motility might provide the mechanism for long-range signalling between the invading hyphal tip and nucleus. For this, we co-expressed a photo-activatable EE marker paGFP-Rab5a (ref. 26) and mCherry-histone-4 in invasive hyphae in *U. maydis*. Following laser-based photo-activation, EEs became visible and travelled rapidly from the hyphal tip towards the nucleus (Supplementary Movie 2 and Fig. 1d, lower panel shows kymograph, in which motility is represented by diagonal lines). We found that 36% of signals (*n*=167) reached or passed the nucleus (Fig. 1e), demonstrating that retrograde EE motility could mediate communication between the hyphal tip and nucleus during infection.

### A synthetic protein blocks specifically EE motility

**Figure 2:**
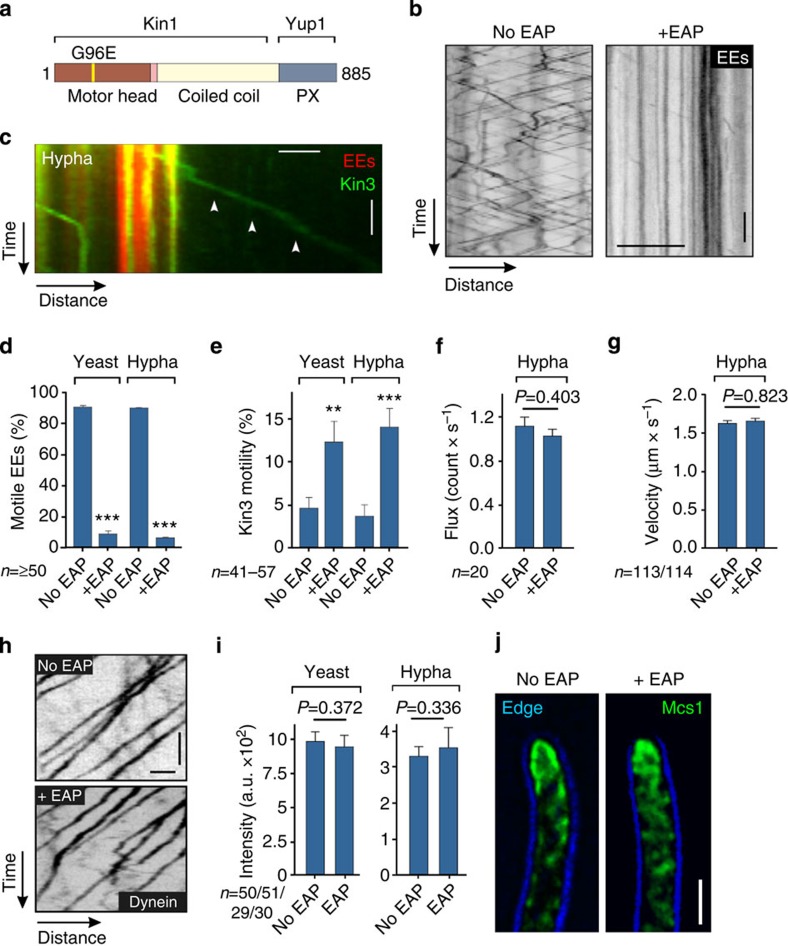
A synthetic anchorage protein blocks fungal EE motility. (**a**) Organization of synthetic linker EAP; N-terminal part of an immobile kinesin-1^rigour^ protein (aa 1-739) fused to an EE-binding Phox domain (PX) of the putative t-SNARE Yup1 (ref. 23). (**b**) Contrast-inverted kymographs showing motility of GFP-Rab5a-labelled EEs. Cells were pre-grown in glucose-containing (no EAP) or arabinose-containing medium (+EAP). The linker blocks EE motility. Scale bars, 3 s (vertical) and 3 μm (horizontal). See Supplementary Movie 3. (**c**) Kymograph showing motility of kinesin-3-GFP (green, arrowheads), unbound to stationary EEs (red) in a hyphal cell expressing EAP for 5 h. Scale bars, 2 s (vertical) and 2 μm (horizontal). See Supplementary Movie 4. (**d**) Motile EEs in yeast-like (yeast) and hyphal cells (hypha) in absence (no EAP) or presence of EAP expression (+EAP). Mean±s.e. and sample size from two experiments is shown. ***Significance at *P*<0.001, unpaired Student’s *t*-test. (**e**) EE-independent movement of GFP-labelled kinesin-3 motors in the presence (+EAP) or absence of EAP (no EAP) in yeast-like cells and hyphae. Mean±s.e. and sample size *n* from two to three experiments is shown. **Significance at *P=*0.0066, ***significance at *P=*0.0004, unpaired Student’s *t*-test. (**f**) Flux of dynein in the presence (+EAP) or absence of EAP (no EAP). Mean±s.e. and sample size *n* from two experiments is shown. No significant difference at *P*=0.4030, unpaired Student’s *t*-test. (**g**) Velocity of retrograde dynein in the presence (+EAP) or absence of EAP (no EAP). Mean±s.e. and sample size *n* from two experiments is shown. No significant difference at *P*=0.823, unpaired Student’s *t*-test. (**h**) Contrast-inverted kymographs showing retrograde motility of dynein in the absence of EAP (no EAP) and after 15 h of expression of EAP (+EAP). Scale bars, 1 s (vertical) and 1 μm (horizontal). (**i**) Apical Mcs1-GFP_3_ signal intensity at the growth region of yeast-like and hyphal cells in the presence (+EAP) or absence of EAP (no EAP). Mean±s.e. and sample size *n* from one experiment is shown. The result was confirmed by a non-quantitative experiment. No significant difference at an error probability of *P*=0.372 and 0.336, unpaired Student’s *t*-test. (**j**) Apical Mcs1-GFP_3_ at the growth region of hyphal cells in the presence (+EAP) or absence of EAP (no EAP). Scale bar, 2 μm.

Bi-directional EE motility in *U. maydis* is driven by the motor protein kinesin-3 (Kin3, ref. 26). We found that a Δ*kin3* mutant was less virulent (Supplementary Fig. 2a,b), suggesting that EE motility is important for infection. However, Kin3 has been implicated in secretion in *U. maydis*^30^ and it is possible, therefore, that inhibition of an EE-independent kinesin-3 function attenuates virulence. To investigate this possibility, we designed a synthetic protein to immobilize EEs more specifically, without blocking kinesin-3 motility. We used an EE anchoring protein (EAP) consisting of a non-motile kinesin-1 motor head^31^, fused to the EE-binding Phox domain of t-SNARE Yup1 (ref. 23; Fig. 2a). The EAP construct was expressed under the inducible *crg1* promoter^32^, which is glucose-repressible and strongly-inducible by arabinose, and transformed into strains expressing GFP-Rab5a-labelled EEs. In the absence of the synthetic protein, normal EE motility was observed (Fig. 2b,d; no EAP), but expression of the chimaeric EAP strongly inhibited EE movement (Fig. 2b,d; +EAP; Supplementary Movie 3). Expression of EAP did not inhibit EE-independent motility of fluorescent Kin3 in yeast or hyphal cells (Fig. 2c,e; Supplementary Movie 4) or fluorescent dynein (Fig. 2f–h), nor did it inhibit apical localization of chitin synthase Mcs1 (Fig. 2i,j), which is dependent on the Kin1 motor^33^. EAP did not therefore affect dynein, kinesin-1 or kinesin-3-mediated transport along microtubules. As these motors mediate the majority of the intracellular transport in *U. maydis*^30^, we conclude that EAP specifically blocks EE motility, but without inhibition of other motor-driven intracellular trafficking.

### EE motility is essential for early plant infection

Having established EAP as a specific tool to block EE motility, we investigated EE motility during early and late plant colonization. EAP was expressed under control of the *crg1*- and plant-induced *mig1* promoter, which is repressed during early plant invasion^34^. We confirmed the expression pattern of both promoters by monitoring cytoplasmic GFP *in planta*. The *crg1* promoter induces GFP expression during early stages of infection and is repressed after 2–3 days, whereas *mig1*-driven expression is observed at 3 days post infection (d.p.i.) onwards (Supplementary Fig. 3).

**Figure 3:**
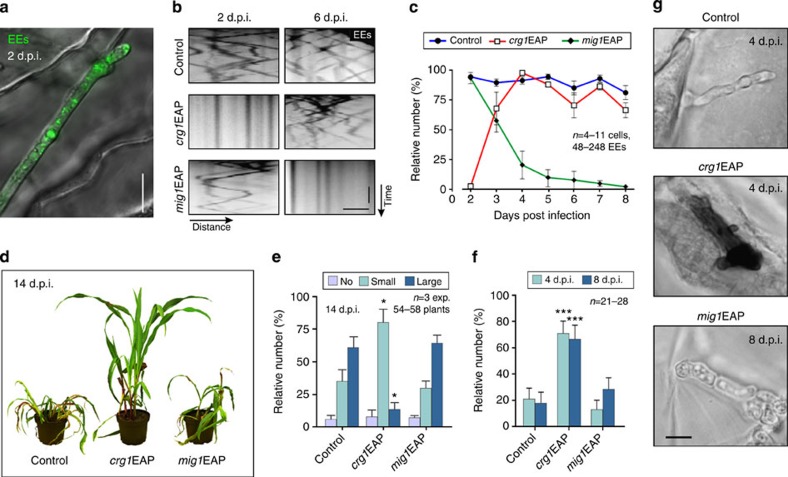
EE motility is essential for early plant infection. (**a**) GFP-Rab5a-labelled EEs in invasive fungal cell (2 d.p.i.). Scale bar, 10 μm. See Supplementary Movie 5. (**b**) Contrast-inverted kymographs showing EE motility in invasive hyphae. Expression of EAP under *crg1* promoter blocks EE motility at 2 d.p.i., whereas *mig1* promoter-driven expression inhibits motility at 6 d.p.i. Scale bars, 3 s (vertical) and 1 μm (horizontal). See Supplementary Movie 6. (**c**) Graph showing EE motility during pathogenic development. Mean±s.e. and sample size *n* from one experiment is shown. The result was confirmed by a non-quantitative experiment. (**d**) Maize infected with wild-type *U. maydis* (control) and mutants that block EE motility during early infection (*crg1*EAP) and late infection (*mig1*EAP) at 14 d.p.i. (**e**) Disease symptoms post infection with control, *crg1*EAP and *mig1*EAP. Mean±s.e.; sample sizes (=number of experiments (exp)) are shown. *Significantly different from control at *P=*0.0468 (small tumours) and *P*=0.0205 (large tumours); unpaired Student’s *t*-test. No tumours: anthocyanin and chlorosis but no swellings; small tumours: swellings <3 mm; large tumours: swellings >3 mm. (**f**) Diaminobenzidine precipitation indicates plant defence-associated H_2_O_2_ in leaves infected with wild type, *crg1*EAP and *mig1*EAP. Mean±s.e. and sample sizes from one experiment is shown. The result was confirmed by a non-quantitative experiment. ***Significantly different from control at *P*=0.0003 (4 d.p.i.) and *P*=0.0009 (8 d.p.i.); unpaired Student’s *t*-test. (**g**) Diaminobenzidine precipitation in control, *crg1*EAP and *mig1*EAP mutants. Scale bar, 5 μm.

To test the role of EE motility in plant infection, we generated SG200 strains, expressing GFP-Rab5a and two mutants that contained EAP under the *crg1* promoter (*crg1*EAP) or *mig1* promoter (*mig1*EAP). SG200 strains are widely used in virulence studies in *U. maydis*^9^,^13^,^29^, because they are able to auto-induces a pheromone cascade, which initiates hyphal growth on charcoal-containing agar medium and during plant infection without the need for fusion of two compatible cells^35^. Using these strains we observed EEs during plant tissue invasion (Fig. 3a and Supplementary Movie 5). In control experiments, EEs moved bidirectionally throughout infection (Fig. 3b,c and Supplementary Movie 5; control). When plants were infected with *crg1*EAP, pre-grown in arabinose-medium, EE motility was blocked for the first 2 days but re-established when the *crg1* promoter was glucose-repressed at later time points (Fig. 3b,c and Supplementary Movie 6). By contrast, EE motility was initially normal in the *mig1*EAP strain, but blocked 3–4 days after infection (Fig. 3b,c and Supplementary Movie 6). Thus, by placing EAP under control of different promoters, we established an infection-stage-specific means of inhibiting EE motility.

We then investigated the role of EE motility during early and late plant infection. Inoculation of maize plants with control strain or *mig1*EAP caused disease symptoms, including inhibition of plant growth (Fig. 3d) and ‘tumour’ formation (Fig. 3e). We therefore conclude that EE motility is not essential at late stages of infection. However, infection with *crg1*EAP led to severe attenuation of disease symptoms (Fig. 3d,e). Blocking EE motility during early infection therefore strongly reduced virulence of the fungus. We reasoned that this might be owing to an inability to suppress plant immunity. One early defence reaction of the plant is to release H_2_O_2_ during an oxidative burst, which can be visualized by precipitation of diaminobenzidine^36^. We stained infected leaves with diaminobenzidine and found little evidence of an oxidative burst in wild-type or *mig1*EAP infections at 4–8 d.p.i. (Fig. 3f,g), which is consistent with suppression of early immune responses during successful *U. maydis* infection^9^,^12^,^13^. By contrast, *crg1*EAP strains induced an oxidative burst (Fig. 3f,g). We conclude that EE motility is required to suppress host defences during early stages of infection.

### Endosome motility is required for fungal effector production

**Figure 4:**
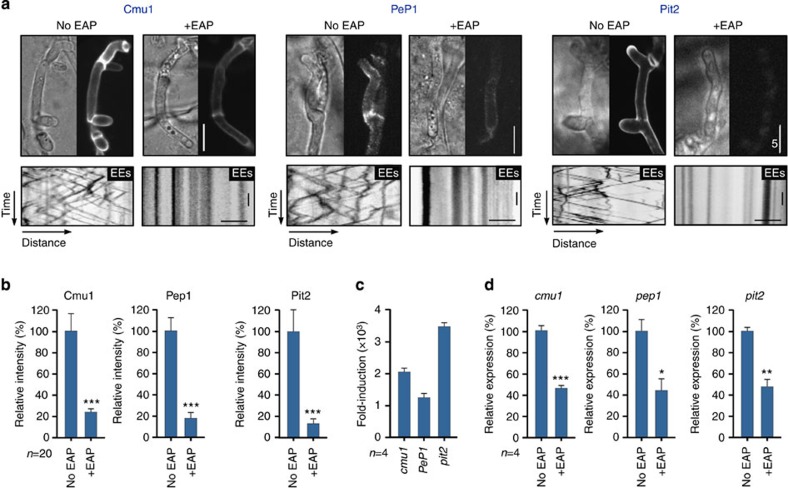
EE motility is required for effector secretion and transcription of effector-encoding genes in *U. maydis* (**a**) Impaired secretion of effectors during infection. Cmu1-mCherry (Cmu1), Pep1-mCherry (Pep1) or Pit2-mCherry (Pit2) expressed in cells that contain EAP, which impaired EE motility (contrast-inverted kymographs, stationary signals appear as vertical lines). Cells were pre-grown in glucose- (no EAP) and arabinose-containing medium (+EAP). Scale bars, 5 μm (upper panels), 3 s (vertical, lower panels) and 2 μm (horizontal, lower panels). (**b**) Secretion of fluorescent protein-effector fusions in plants at 2 d.p.i. Mutants were pre-grown in glucose- (no EAP) and arabinose-containing medium (+EAP). Mean±s.e. and sample size from one experiment is shown. The result was confirmed by a non-quantitative experiment. ***Significance at error probability *P*_cmu1_ and *P*_pit2_*=*0.0002*, P*_pep1_<0.0001; unpaired Student’s *t*-test. Signals were corrected by subtracting intracellular fluorescent background. (**c**) Induction of fungal effector transcription during in plant infection. Expression in planta at 1 d.p.i. was compared with transcript levels in liquid culture. Mean±s.e. and sample sizes (= number of experiments) are shown. (**d**) Relative transcript levels of *cmu1, pep1* and *pit2* genes in infectious fungal cells grown at 1–2 d.p.i. Cells were pre-grown in glucose- (no EAP) and arabinose-containing medium (+EAP). See Supplementary Methods section. Expression of effector genes in control cells was set to 100%. Mean±s.e. and sample sizes (=number of experiments) are shown. *Different from control at *P*_pep1_*=*0.0149, **different from control at *P*_pit2_*=*0.0026 and ***different from control at *P*_cmu1_*=*0.0009; unpaired Student’s *t*-test.

Suppression of plant immunity responses suggested the EE motility may be necessary for fungal effector production and secretion. To test this idea, we expressed functional gene fusions of mCherry and Cmu1 (ref. 13), the immunity suppressor Pep1 (ref. 9) and the protease inhibitor Pit2 (ref. 12), and observed their secretion *in planta*. Fungi expressing Cmu1-mCherry, Pep1-mCherry caused strong disease symptoms (Supplementary Fig. 1a,b), indicating that the fusion protein is fully functional. In contrast, Pit2-mCherry producing strains showed attenuated virulence (Supplementary Fig. 1a,b). Each effector accumulated in the apoplastic space (Supplementary Fig. 1c–e), as previously reported^9^,^12^,^13^. We then infected plants with *crg1*EAP strains co-expressing each fluorescent effector protein and the EE marker GFP-Rab5a. When cells were pre-grown in glucose-containing media, infecting fungi showed EE motility and normal secretion of fluorescent effectors (Fig. 4a, left panels; lower left kymographs show EE motility by diagonal lines). When cells were grown in inducing (arabinose-containing) media, EAP was produced and EE motility was inhibited (Fig. 4a, right panels; lower right kymograph shows the absence of motility). Importantly, in these cells, the peripheral signal of all three effector proteins was decreased markedly (Fig. 4a,b and Supplementary Fig. 1c–e), suggesting that a block in EE motility impairs effector secretion. This defect in effector secretion raised the possibility that retrograde EE-based signalling is required to induce effector gene transcription at the nucleus. We therefore measured transcript levels of *cmu1*, *pep1* and *pit2* using quantitative real-time PCR (qRT–PCR). None of the effectors was expressed in axenic culture, whereas effector expression was strongly induced *in planta* when EAP was not present (Fig. 4c; bars show induction compared with liquid culture). However, when EE motility was blocked by expression of EAP under the *crg1* promoter, we observed significantly reduced effector transcription (Fig. 4d, +EAP; error probability *P*_cmu1_: 0.0009; *P*_pep1_: 0.0149 and *P*_pit2_: 0.0026; unpaired Student’s *t*-test). We conclude that effector transcription and, consequently, effector secretion depends on EE motility.

**Figure 5:**
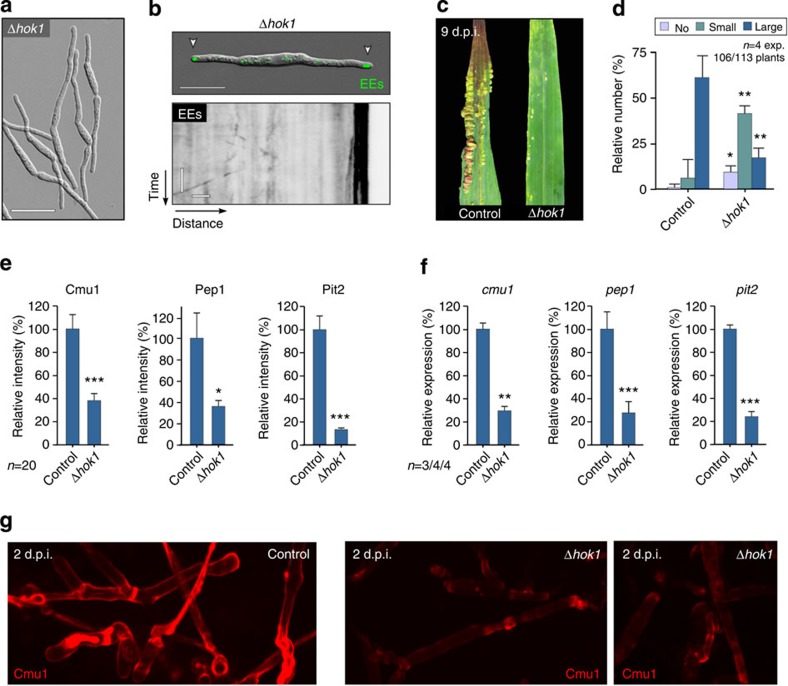
Effector secretion and pathogenicity are impaired in *hok1* null mutants. (**a**) Phenotype of *hok1* deletion mutants. Scale bar, 20 μm. (**b**) EE localization and motility in *hok1* deletion mutants. Upper panel shows EE clusters (GFP-Rab5a, green, arrowheads) at the poles of the short mutant cells. Lower kymograph shows the absence of EE motility in Δ*hok1* mutants. Scale bars, 20 μm (upper panel), 3 s (vertical, lower panel) and 1 μm (horizontal, lower panel). (**c**) Plant disease symptoms at 9 days after infection of maize plants with control and Δ*hok1* strains. (**d**) Plant symptoms at 14 days after infection with control and *hok1* deletion mutants. Mean±s.e. and sample sizes *n* (=number of experiments (exp)) are shown. *Different from control at error probability *P*=0.019; **different from control at error probability *P*=0.0047; unpaired Student’s *t*-test. (**e**) Secretion of fluorescent effectors, indicated by the signal intensity at the periphery of the invasive hyphae in plants at 2 d.p.i. Mean±s.e. and sample sizes from one experiment are shown. The result was confirmed by a non-quantitative experiment. *Different from control at *P*_pep1_*=*0.0184, ***different from control at *P*_cmu1_*=*0.0002 and *P*_pit2_*<*0.0001; unpaired Student’s *t*-test. (**f**) Relative transcript levels of *cmu1, pep1* and *pit2* in cells grown in infected plant tissue at 1–2 d.p.i. Effector expression in infecting control strains was set as 100%. Mean±s.e. and sample sizes *n* (=number of experiments) are shown. **Significance at error probability *P*_cmu1_*=*0.0021 and *P*_pep1_*=*0.0096; ***significance at error probability *P*_pit2_<0.0001; unpaired Student’s *t*-test. (**g**) Secretion of Cmu1-mCherry in control and Δ*hok1* mutants. Note that secretion of the effector in Δ*hok1* mutants is reduced compared with control strain. Scale bar, 5 μm.

**Figure 6:**
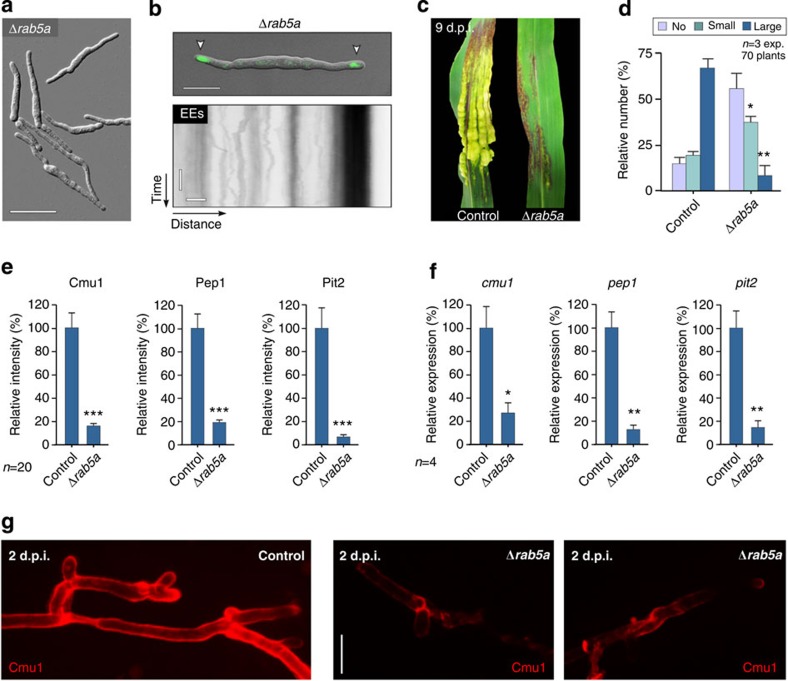
Effector secretion and pathogenicity are impaired in *rab5a* null mutants. (**a**) Phenotype of *rab5a* deletion mutants. Scale bar, 20 μm. (**b**) EE localization and motility in *rab5a* deletion mutants. Upper panel shows EE clusters (Yup1-GFP, green, arrowheads) at the poles of the short mutant cells. Lower kymograph shows the absence of EE motility in Δ*rab5a* mutants. Scale bars, 3 μm (upper panel), 5 s (vertical, lower panel) and 2 μm (horizontal, lower panel). (**c**) Plant disease symptoms at 9 days after infection of maize plants with control and Δ*rab5a* strains. (**d**) Plant symptoms at 14 days after infection with control and *rab5a* deletion mutants. Mean±s.e. and sample sizes *n* (=number of experiments (exp)) are shown. *Different from control at error probability *P*=0.019; **different from control at error probability *P*=0.0047; unpaired Student’s *t*-test. (**e**) Secretion of fluorescent effectors indicated by the signal intensity at the periphery of the invasive hyphae in plants at 2 d.p.i. Mean±s.e. and sample size *n* from one experiment is shown. The result was confirmed by a non-quantitative experiment. ***Significance at error probability *P*<0.0001; unpaired Student’s *t*-test. (**f**) Relative transcript levels of *cmu1, pep1* and *pit2* in cells grown in infected plant tissue at 1–2 d.p.i. Effector expression in infecting control strains was set as 100%. Mean±s.e. and sample sizes *n* (=number of experiments) are shown. *Significance at error probability *P*_cmu1_*=*0.0257, **significance at error probabilities *P*_pep1_*=*0.0083 and *P*_pit2_*=*0.007; unpaired Student’s *t*-test. (**g**) Secretion of Cmu1-mCherry in Control and Δ*rab5a* mutants. Note that secretion in Δ*rab5* is reduced, compared to the control strain. Scale bar, 5 μm.

Many signalling pathways are located to EEs, including phosphatidylinositol 3-phosphate (PtdIns3P) signalling^19^. The Phox domain of EAP is expected to bind to PtdIns3P, and thus might have unrecognized side effects. We therefore set out to find EAP-independent ways of blocking EE motility and the putative retrograde signalling mechanism. Recently, the orthologous hook proteins Hok1 and HookA, identified, respectively, in *U. maydis*^37^ and *Aspergillus nidulans*^38^, were shown to act as adaptors that link microtubule-dependent motors to EEs. They are thus crucial for fungal EE motility, and we set out to investigate effector transcription and secretion in Δ*hok1* mutants. We found that *hok1* null mutants were still able to generate hyphal cells (Supplementary Fig. 4), demonstrating that pheromone signalling pathways were not affected in this strain. Consistent with previous reports, these hyphae remained short, and we observed immobile EEs clustered at their cell ends (Fig. 5a,b) (ref. 37). Similar to EAP-expressing strains, *hok1* null mutants were impaired in their ability to cause disease (Fig. 5c,d). Most strikingly, the transcription and secretion of all tested effectors was reduced significantly in *hok1* null mutants (Fig. 5e–g). These results are therefore consistent with our conclusion that EE motility is required for the induction of effector production and exocytosis. To test this hypothesis further, we deleted the fungal homologue of the small GTPase Rab5 (ref. 39), which is of central importance for EE function in mammalian cells^40^. In the absence of Rab5a, short hyphae formed (Fig. 6a), containing largely immobile EE accumulations at the ends of cells (Fig. 6b), which were labelled by the EE marker Yup1-GFP (ref. 23). Similar to Δ*hok1* mutants, *rab5a* null mutants caused only mild pathogenicity symptoms (Fig. 6c,d), and, again, transcription and secretion of all tested effectors were significantly reduced (Fig. 6e–g). We conclude that inhibition of EE motility, either by expression of EAP, deletion of the motor adaptor Hok1 or deletion of *rab5a*, reduces effector transcription and secretion, thereby preventing the fungus from successful colonization of plant tissue.

### An endosome-associated MAPK controls effector transcription

Our observations indicated that Rab5a-positive EEs mediate retrograde signaling that initiates transcription and subsequent secretion of effectors. We therefore set out to identify potential signalling components in this pathway. In mammalian cells, purified Rab5-positive EEs contain numerous long-range signalling proteins. Among these are MAPKs of the extracellular signal-regulated kinase (ERK) signalling pathway, including B-Raf (B-rapidly accelerated fibrosarcoma), MEK1 (MAPK or ERK kinase), ERK1/2 (ERKs 1 and 2) and kinase p38 (MAPK p38; overview in ref. 21). In addition, the GTPases Rap1 (Ras-related protein 1), the adaptor protein Gab2 (growth factor receptor-bound protein 2; ref. 18) and the EE-associated adaptor protein, phosphotyrosine interaction, PH domain and leucine zipper containing 1 (APPL1; ref. 41) are involved in signalling. Finally, components of the Akt signalling pathway, such as glycogen synthase kinase-3 beta (GSK3β; ref. 42) and the transforming growth factor beta (TGF-β)/smad signalling pathway, including the signalling mediators SMAD2 and SMAD4 (ref. 43), the FYVE-domain containing endofin^44^ and the SMAD anchor for receptor activation (SARA; ref. 45) transport signals from the plasma membrane to the nucleus (overview in refs 19, 21). We used the sequences of these human proteins (see Methods section for accession codes) in a reciprocal BLAST search and screened the published genome sequence of *U. maydis* for fungal orthologues of any of these signalling proteins. We did not find APPL-like proteins or components of the SMAD/SARA pathway nor did we identify homologues of Gab2 or Rap1. However, we found a GSK3β homologue (64% identical to human GSK3β; NCBI accession code: XP_756707.1) and the MAPK Kpp2/Ubc2 (refs 46, 47; NCBI accession code: XP_759452.1; 49% identical to human ERK1 and 54% identical to human MAPK p38 and ERK2) and the MAPK kinase kinase Kpp4/Ubc4 (refs 48, 49; 37% identical to human MEK1). *U. maydis* contains a single MAPK module^49^. This consists of the MAPKK kinase Kpp4/Ubc4 (refs 48, 49), the downstream MAPK Fuz7 (ref. 50) and the MAPKs Kpp2/Ubc2 (refs 46, 47), Kpp6 (ref. 51) and Crk1 (refs 52, 53 and Supplementary Fig. 5a). All MAPK module components are required for full virulence in *U. maydis*^46^,^47^,^49^,^50^,^51^,^52^ and thus could be involved in virulence-associated signalling. We therefore fused single GFP to all these kinases and to the GSK3β homologue, and asked whether any of these show long-range motility within hyphal cells. In these experiments, the MAPK Crk1 showed constant and directed movements. The MAPKK kinase Kpp4 and GSK3β homologue occasionally moved, whereas the MAPK kinase Fuz7 and the MAPKs Kpp2 and Kpp6 were not motile (Supplementary Fig. 5b). Interestingly, all moving kinases were also concentrated in the fungal nucleus (Supplementary Fig. 5c), which supports the notion that they serve roles in membrane-to-nucleus signalling.

**Figure 7:**
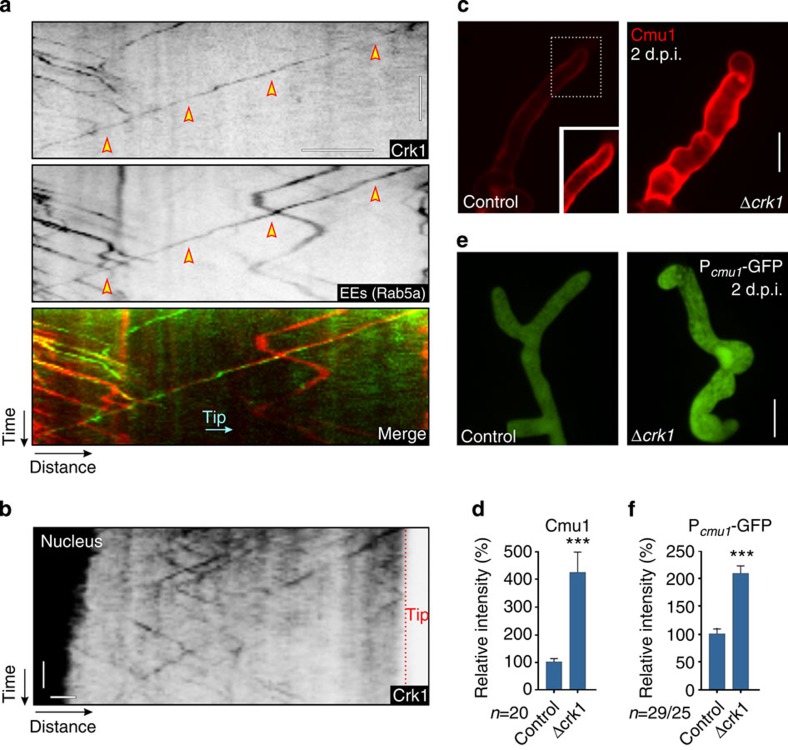
EE-associated localization of the MAPK Crk1 and its role in effector secretion and transcription. (**a**) Kymographs showing motility of Crk1-GFP_3_ (Crk1) on EEs (Rab5a, arrowheads). Direction towards the hyphal tip is indicated (Tip). Note that the endogenous copy of *crk1* was fused to a triple-GFP tag. Motility was observed in a photo-bleached area. The upper two kymographs are contrast inverted. Scale bars, 5 s (vertical) and 5 μm (horizontal). See Supplementary Movie 7. (**b**) Kymographs showing motility of Crk1-GFP_3_ in a fungal hypha during invasion of plant tissue. Note that trajectories of Crk1-GFP_3_ signals are discontinuous, as signals move in and out of the confocal focus. Scale bars, 3 μm (upper panel), 5 s (vertical, lower panel) and 2 μm (horizontal, lower panel). See Supplementary Movie 8. (**c**) Secretion of fluorescent Cmu1 effector in control and *crk1* null mutants (Δ*crk1*). Both images were scaled identically to show different degree of secreted Cmu1-mCherry. Inset shows Cmu1-mCherry after scaling the control images. Scale bar, 5 μm. (**d**) Secretion of fluorescent Cmu1 indicated by the signal intensity at the periphery of the invasive control and Δ*crk1* mutant hyphae in plants at 2 d.p.i. Mean±s.e. and sample size *n* from two experiments is shown. ***Significance at error probability *P*=0.0005; unpaired Student’s *t*-test. (**e**) Expression of cytoplasmic GFP under the *cmu1* promoter in invasive control and Δ*crk1* mutant hyphae in plants at 2 d.p.i. Both images were scaled identically. Scale bar, 5 μm. (**f**) Expression of cytoplasmic GFP under the *cmu1* promoter in invasive control and Δ*crk1* mutant hyphae in plants at 2 d.p.i. Mean±s.e. and sample size *n* from two experiments is shown. ***Significance at error probability *P*<0.0001; unpaired Student’s *t*-test.

In the initial localization experiments, very faint Crk1-GFP signals moved over long distances. To test whether Crk1 is transported via retrograde moving EEs, we fused the endogenous copy of *crk1* to a triple-GFP tag and co-expressed the fusion protein with endosomal mCherry-Rab5a in a strain that forms hyphal cells in liquid culture. We observed that Crk1-GFP_3_ localized to rapidly moving Rab5a-positive EEs (Fig. 7a and Supplementary Movie 7). Retrograde Crk1-GFP_3_ signals travelled for up to 30 μm (Fig. 7a), which is consistent with the notion that the MAPK could participate in retrograde signalling to the nucleus during plant infection. To test this idea further, we expressed Crk1-GFP_3_ in the solo-pathogenic strain SG200 and investigated Crk1 motility *in planta*. We found that the MAPK also travelled over long distances in invasive hyphae and localized in the nucleus (Fig. 7b and Supplementary Fig. 6a). This result suggests that Crk1 could participate in transmitting signals from the hyphal tip to the nucleus, thereby controlling effector transcription and secretion. We addressed this possibility by deleting *crk1* in a strain that expressed Cmu1-mCherry. Surprisingly, we found that effector secretion was significantly increased in the absence of Crk1 (Fig. 7c, both images are identically processed; Fig. 7d), suggesting an inhibitory signalling function. We therefore set out to test whether expression of *cmu1* was induced by deletion of the MAPK *crk1*. The resultant *crk1* null mutants, however, showed a defect in morphology and were significantly impaired in penetration of the plant. Consequently, RT–PCR analysis of *cmu1* could not be reliably carried out because of the small proportion of fungal hyphae that successfully penetrated host tissue, which would normally express *cmu1* specifically. To circumvent this problem, we made a transcriptional fusion of cytoplasmic GFP under the control of the *cmu1* promoter (*P*_cmu1_) and quantitatively analysed the GFP signal intensity in control hyphae at 1 d.p.i., when the fungus had not yet invaded the plant, and 2 d.p.i., when fungal cells were found inside the plant tissue. We found only traces of cytoplasmic GFP at 1 d.p.i., but a strong fluorescent signal when the fungus had penetrated the plant (Supplementary Fig. 6b,c). This is consistent with the induction of *P*_cmu1_ during plant invasion. Significantly, less fluorescence was detected in Δ*hok1* mutants (Supplementary Fig. 6b,c), which is consistent with the role of EE motility in effector transcription. We next generated a *crk1* null mutant, expressing the *P*_cmu1_-GFP construct. We infected plants and visualized infection structures after 2 d.p.i. Interestingly, we found a significant increase in cytoplasmic GFP fluorescence, indicating that *P*_cmu1_ is more strongly induced in Δ*crk1* mutants (Fig. 7e,f). When considered together, these results provide evidence that the EE-associated MAPK, Crk1, acts as a repressor of effector transcription.

## Discussion

An increasing body of evidence suggests that pathogenic fungi and oomycetes secrete a large repertoire of effector proteins into plant cells to suppress plant immunity and facilitate biotrophic growth^1^,^3^,^4^,^5^,^8^,^9^,^10^,^11^,^12^,^13^,^29^. In this report, we provide evidence that motility of EEs is essential for effector production and subsequent secretion by the filamentous fungal pathogen *U. maydis*. This conclusion is supported by several independent experimental strategies. First, we generated an artificial anchor protein EAP that consists of a mutated kinesin-1 motor head that tightly binds to microtubules^31^ and the C-terminal Phox domain of the SNARE Yup1 (ref. 23). Phox domains bind to the lipid PtdIns3P (ref. 54), which is, itself, enriched in the membrane of EEs^55^. Strong expression of the chimaeric EAP almost abolished EE motility and significantly reduced the secretion of three tested effector proteins into the apoplastic space between the fungus and the plant cell. One could argue that the presence of EAP simply blocks microtubule-dependent delivery of effector proteins. We consider this possibility unlikely, however, because microtubule-based membrane trafficking in *U. maydis* depends on kinesin-1, kinesin-3 and dynein^30^, and EAP expression did not impair motility of fluorescent kinesin-3 or dynein nor did it affect delivery of chitin synthase Mcs1 to the growing tip of the cell, a process depending on kinesin-1 (ref. 33). We conclude from these results that EAP specifically blocks EE movement but does not affect other membrane trafficking pathways. Consequently, we consider it very likely that impaired effector secretion is a consequence of reduced EE motility. This notion is further supported by the observation that *hok1* and *rab5a* null mutants also show almost identical defects in effector secretion. It was recently reported that *hok1* null mutants have a defect in motor-to-cargo coupling, which results in a severe reduction in EE transport^37^. A similar defect is seen in *rab5a* null mutants, although these cells are most likely impaired in various EE functions, because this GTPase is of central importance for motility, identity and function of these organelles^40^. However, when taken together, all three mutant lines (EAP-expressing, *hok1* and *rab5a* null mutants) show very significant reductions in secretion of the three tested effectors. Motile EEs therefore appear to be essential during the interaction of the fungal pathogen and the plant cell.

**Figure 8:**
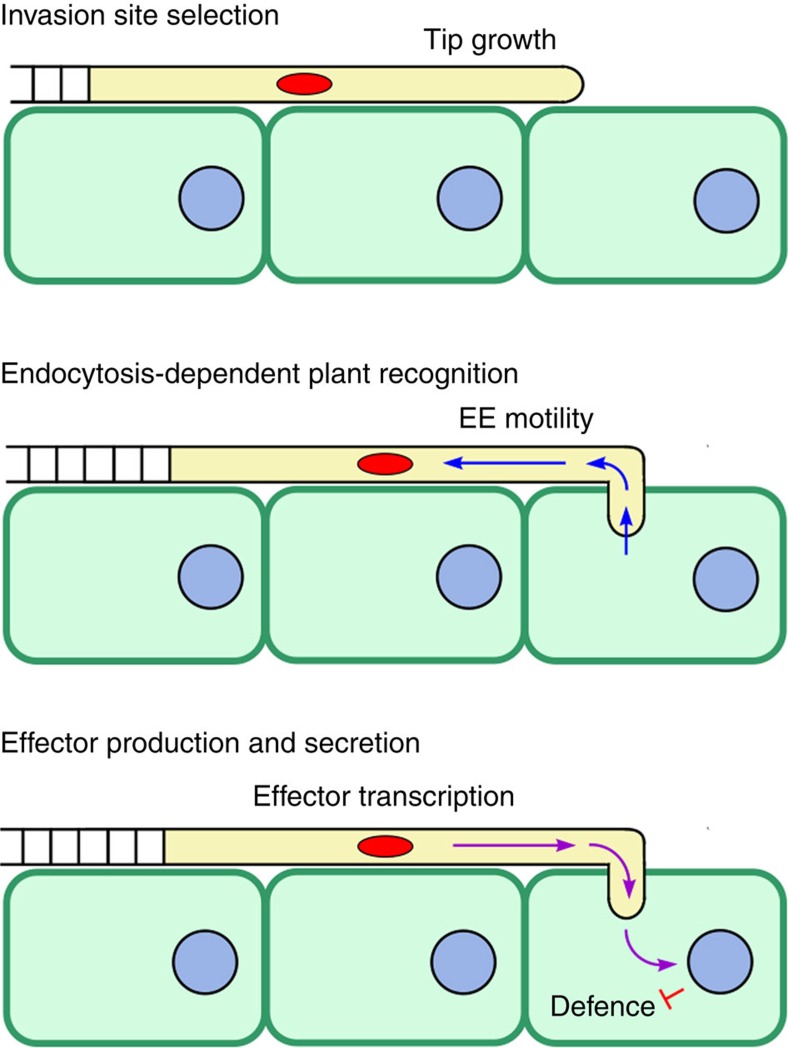
Model for retrograde EE motility in effector-mediated plant invasion by pathogenic fungi. The invading hyphal tip perceives plant cues during the initial stages of infection. Endocytic uptake relays this information to motile EEs that transport the signal to the nucleus. This retrograde signalling mechanism triggers effector gene expression. Subsequently, effectors are secreted at the hyphal tip to suppress plant immunity and facilitate fungal invasion.

How then do moving EEs support effector secretion during plant infection? We recognized that elongated fungal cells face a considerable challenge during host infection in communicating between the invading distal tip and the nucleus. This is clearly necessary in order to produce effector proteins specifically during the very early stages of infection. In *U. maydis*, the nucleus is, for instance, ~22 μm behind the invading hyphal tip. In the crowded cytoplasm, even a few micrometres pose an insurmountable problem because passive diffusion is severely hindered^56^. This suggests that communication between the hyphal tip and nucleus involves an active transport-dependent signalling mechanism. In mammalian neurons, such long-range signalling involves EEs, which travel along microtubules, delivering signalling components from the plasma membrane to the nucleus^19^,^20^,^21^. Long-range and microtubule-dependent motility of EEs have been reported in *U. maydis*^23^,^26^ and other fungi^24^,^25^ (overview in ref. 22), and we have previously speculated that moving fungal EEs might participate in retrograde signalling^57^. The evidence provided here is consistent with such a function. The observed defects in effector secretion, for example, are most likely owing to impaired effector gene transcription as a consequence of blocked EE-dependent retrograde signalling (Fig. 8). Thus, EE motility acts at the level of transcription, which implies a novel, endosome-based signalling mechanism that mediates communication between the fungal nucleus and invading hyphal tip, and which is essential for fungal pathogenicity.

A role for fungal EEs in long-range signalling has not hitherto been described. Indeed, our understanding of the signalling pathways associated with fungal EEs is in its infancy. In mammalian cells, several signalling pathways are located on EEs that include the MAPKs of the ERK signalling pathway^19^,^21^. Our search for homologues of retrograde signalling components, associated with EEs in humans, revealed known components of a MAPK module. This included the MAPK Kpp2/Ubc2, which is shown to be involved in pathogenic development^46^,^47^, and therefore is a good candidate for EE-associated signalling. However, Kpp2 did not show directed movement, whereas the MAPK Crk1 was transported by EEs over long distances. This observation, coupled with localization of Crk1 in the fungal nucleus, and de-repressed effector transcription and secretion, strongly suggests that Crk1 participates in long-range EE-based signalling. We also find the MAPK kinase Fuz7 and the MAPKK kinase Kpp4 in the nucleus, and at least Kpp4 showed occasional motility. Therefore, we consider it most likely that the MAPK module in *U. maydis* delivers signals from the plasma membrane into the nucleus. Crk1 appears to be of key importance in this pathway, which adds to the known roles of Crk1 in morphogenesis^52^ and mating^53^.

Surprisingly, *crk1* null mutants showed increased expression and secretion of effector protein. This suggests that Crk1 is a negative regulator that represses effector transcription. We speculate that it might therefore counteract an unknown EE-bound phosphatase to collectively fine-tune expression of effector genes during infection. Such balanced activities of phosphatases and kinases mediate many essential cellular processes, including cell-cycle progression^58^, axonal migration and transport^59^,^60^, and various signalling pathways^61^. Finally, it is worth mentioning that Gsk3β also showed directed movement in the cell. Mammalian Gsk3β is a direct target of the serine/threonine kinase Akt (ref. 62), and both dynamically interact with the Rab5-effector APPL1 on EEs^42^. *U. maydis* does not contain an APPL homologue, and whether the Gsk3β homologue is associated with fungal EEs is not yet clear. However, the identification of an EE-associated and MAPK-based signalling pathway in effector production raises the possibility that EEs have more widespread roles in the plasma membrane-to-nucleus signalling than has hitherto been recognized in pathogenic fungi.

## Methods

### Strains and plasmids

All *U. maydis* strains were constructed in the genetic background of AB33 (ref. 63) or SG200 (ref. 35; Table 1). To observe EEs motility during plant infection, plasmid poGRab5a (ref. 64) was integrated ectopically into strain SG200, resulting in SG200GRab5a. Strains AB33mChRab5a and AB33Kin3G_mChRab5a were generated by integrating the plasmid po_m_ChRab5a into AB33 and AB33Kin3G (ref. 26), respectively. SG200ΔKin3 and SG200ΔHok1 were generated by deleting the endogenous *kin3* or *hok1* gene using pΔKin3 (ref. 27) or p^H^ΔHok1 (ref. 37), respectively. SG200Cmu1Ch_ΔHok1, SG200Pep1Ch_ΔHok1 and SG200nPit2Ch_ΔHok1 were generated by deleting the endogenous *hok1* gene using p^N^ΔHok1 (ref. 37). Deletions were confirmed by Southern blot. Ectopic integration of plasmid pCoGRab5a (ref. 65) resulted in SG200ΔKin3_GRab5a. All plasmids were generated by standard cloning procedures or *in vivo* recombination in the yeast *Saccharomyces cerevisiae*^66^. Detailed cloning information is provided in the Supplementary Methods.

### Growth conditions

All cultures of *U. maydis* strains were grown overnight at 28 °C in either YEPS_light_ medium (1.0% yeast extract, 0.4% peptone and 0.4% sucrose) or complete medium (CM), containing 1% (w/v) glucose (CM_glc_) or 1% (w/v) arabinose (CM_ara_) at 200 r.p.m. Hyphal growth was induced by transferring cells, grown in CM_glc_, into nitrate minimal (NM) medium supplemented with 1% (w/v) glucose. Cells were grown under these conditions for 5–10 h at 200 r.p.m. (28 °C).

### Microscopic techniques

Spinning disc confocal microscopy of infected plant leaves was performed using a VisiScope Confocal Cell Explorer (Visitron System, Munich, Germany) that consisted of an IX81 motorized inverted microscope (Olympus, Hamburg, Germany), a CSU-X1 Spinning Disc unit (Yokogawa, Tokyo, Japan), a PlanApo UPlanSApo × 63/1.35 oil objective (Olympus, Hamburg, Germany) and a Photometrics CoolSNAP HQ2 camera (Roper Scientific, Germany). The fluorescent tags and dyes were excited using a VS-LMS6 Laser Merge System with two solid-state lasers (488 nm/100 mW and 561 nm/100 mW, Visitron System) and a 405-nm/100-mW diode laser, which was 50/50 split. For photo-activation studies, the 405-nm/100-mW diode laser split and directed into a 2D-VisiFRAP Realtime Scanner (Visitron System). Co-observation of GFP and RFP was done using an OptoSplit II LS Image Splitter (Cairn Research Limited, Faversham, UK). All parts of the system were under the control of the software package VisiView (Visitron System).

Laser-based epi-fluorescence microscopy of cultured cells was done as previously described^27^. In brief, cells were placed onto a 2% (w/v) agar cushion and immediately observed using an IX81 motorized inverted microscope, equipped with a PlanApo × 100/1.45 Oil TIRF (Olympus, Hamburg, Germany). A VS-LMS4 Laser-Merge-System with solid-state lasers (488 nm/50 mW or 75 mW and 561 nm/50 mW or 75 mW; Visitron System) was used to excite the fluorescent protein tags. Photo-bleaching experiments were done using a 405-nm/60-mW diode laser, which was dimmed by an ND 0.6 filter, resulting in 15 mW output power, coupled into the light path by an OSI-IX 71 adaptor (Visitron System). The system was regulated by a UGA-40 controller (Rapp OptoElectronic GmbH, Hamburg, Germany) and a VisiFRAP 2D FRAP control software for MetaSeries 7.5.1 (Visitron System). Dual imaging of GFP and RFP was carried out using a Dual-View Micro imager (Photometrics, Tucson, USA) equipped with a dual-line beam splitter (z491/561, Chroma, Rockingham, USA) with an emission beam splitter (565 DCXR, Chroma, Rockingham, USA), an ET-Bandpass 525/50 (Chroma, Rockingham, USA) and a BrightLine HC 617/73 (Semrock, Rochester, USA). A Photometrics CoolSNAP HQ2 camera was used to capture all images. All parts of the system were under the control of the software package MetaMorph (MDS Analytical Technologies, Winnersh, UK).

Scanning electron microscopy was performed using yeast-like cells of strain SG200ΔHok1, which grown on CM agar supplemented with 1% glucose and 1% charcoal for 5 days at 28 °C. Images were acquired using Nikon D5000 camera that was connected to the Nikon stereomicroscope SMZ800 (Nikon, Kingston, Surrey, UK). The 10-mm diameter agarose discs containing yeast-like and hyphal cells were attached to a cryostage and rapidly frozen in liquid nitrogen slush, followed by water sublimation at −95 °C for 3 min in Jeol JSM-6390LV scanning electron microscope (JEOL, Ltd, Welwyn Garden City, UK). This was followed by gold sputter coating using an Alto 2100 chamber (Gatan Ltd., Oxfordshire, UK) and observation in a Jeol JSM-6390LV scanning electron microscope (JEOL, Ltd).

### Quantitative analysis of motility *ex planta*

EE motility in the presence or absence of EAP was measured in cells grown in NM_glu_ (no EAP) and NM_ara_ (+EAP, expression induced for 5 h). GFP-Rab5a signals that did not move for 10 s were considered as non-motile EEs. The effect of EAP on flux of fluorescent dynein was measured within photo-bleached hyphal cells, beginning 5 μm behind the tip and extending for 20 μm in NM_glu_ (15 h) and NM_ara_ (expression induced for 15 h). Motility was analysed in kymographs. Kinesin-3 motility was measured in photo-bleaching apical parts of yeast-like and hyphal cells, using a 405-nm/60-mW diode laser, dimmed to 15 mW output using kymographs. Only signals that did not co-localize with mCherry-Rab5a were counted and compared with all kinesin-3-GFP signals, irrespective of co-localization with EEs. The degree of secretion of Mcs1-GFP_3_ was estimated by measuring the average intensity of the GFP signal in the apical parts of cells forming a medium-sized bud. The intensity values were corrected by the cytoplasmic background.

### Fungal plant infection

*U. maydis* strains were grown in either YEPS_light_, CM_glc_ or CM_ara_ to an OD_600_ of 0.8–1.0. Cells were harvested by centrifugation and re-suspended in water. A quantity of 0.2 or 0.5 ml of this cell suspension (OD_600_~1.0) were injected into 10-day-old maize plants (Golden Bantam; Chase Organics Ltd., Hersham, Surrey, UK). Pathogenicity assays were performed as described previously^67^. In brief, infected plants were grown in a GroBank (CSF Plant Climatics, Wertingen, Germany; 16 h light, 8 h darkness, 28 °C). Eight- to nine-day-old plants were infected by injection of 0.5 ml *U. maydis* cells, pre-grown to OD_600_=0.8 and washed with water into the stem of the plant. Disease symptoms were scored at 9–14 days after infection. The scoring criteria were as follows: ‘no tumours’ (chlorosis and antocyanin formation), ‘small tumours’ (chlorosis, antocyanin formation and tumours up to 3 mm) and ‘large tumours’ (chlorosis, antocyanin formation and tumours over 3 mm). All infection assays were done in at least three experiments and compared by Student’s *t*-test by using the software Prism 4.03 (GraphPad Software Inc., La Jolla, CA, USA).

### Microscopy of infected plant tissue

Propidium iodide/wheat germ agglutinin-AF488 and diaminobenzidine (Sigma-Aldrich, UK) staining in infected plant leaves was done as described previously^67^. In brief, infected leaf sections were incubated in staining solution (1 mg ml^−1^ diaminobenzidine, 0.05% (v/v) Tween 20 and 10 mM Na_2_HPO_4_) under mild vacuum for 60 min, followed by 4 h shaking at 80–100 r.p.m. at room temperature. Samples were incubated in ethanol:acetic acid:glycerol (3:1:1) and stored in fresh de-stain solution at 4 °C (at 90 °C for 15 min). Before microscopy, samples were washed in PBS, pH 7.4, and microscopically analysed.

To visualize nucleus and effector secretion in early stages of infection, plants were infected with the strain SG200G_3__NLSR_3_ and SG200Cmu1Ch_G_3_ (for experimental usage of strains, see Supplementary Table 2). An ~1 × 1-cm region at ~5 cm beneath the infection point was taken from the third inner leaf and placed on Carolina observation Gel (Carolina Biological Supply Company, Burlington, USA). The invasion site was identified by bright-field microscopy, followed by acquiring Z-stacks capturing red and green fluorescence, using an OptoSplit II LS Image Splitter. Z-stacks covered 30 μm at 0.2 μm step size (exposure time of 200 ms, binning 1 and the 488-/561-nm laser at 20%/50%). After merging stacks, maximum projections and three-dimensional reconstructions were done using MetaMorph (Molecular Devices, Winnersh, UK).

EE motility inside invading fungal cells was measured after infection of maize plants with strain SG200GRab5a. Leaf samples were collected at several time points post infection, and GFP-Rab5a motility was recorded in randomly selected invasive hyphal cells. The proportion of moving EEs was determined in movies, covering 8–10 s observation time.

To analyse EE motility in fungal cells at 1 d.p.i., infected leaf tissue was prepared for microscopic analysis. The samples were incubated in 2 μg ml^−1^ Calcofluor solution (F3543-Fluorescent Brightener 28, Sigma, UK) for 30 s, water rinsed and observed on Carolina Observation Gel, using a spinning disc confocal microscope. Invasion sites were identified and Z-stacks were generated as described above. Calcofluor was excited using a 405-nm laser at 15% output power. In photo-activation experiments, paGFP-Rab5a was excited at the invasion site using a focussed 405-nm laser at 15% output power, followed by immediate acquisition of 150 planes using the OptoSplit II LS Image Splitter (Cairn Research Limited), and the 488- and 561-nm observation lasers at 30% and 50% output power, respectively. Motility of photo-activated EEs was analysed in kymographs by using MetaMorph.

To test for *crg1* or *mig1* promoters activities *in planta*, maize plants were infected with 0.5 ml suspension of strain SG200*crg*G and SG200*mig*G, and pre-grown in CM_ara_ or CM_glc_. Maize tissue samples were collected and observed using an IX81 microscope and a VS-LMS4 Laser Merge System, the 488-nm observation laser at 100% output power at an exposure time of 150 ms and image binning 1.

Quantitative analysis of fluorescent intensity of Cmu1-mCherry, Pep1-mCherry and Pit2-mCherry was done by measuring the average fluorescent intensity at the periphery of hyphal cells of strains SG200GRab5a_cEAP_Cmu1Ch, SG200GRab5a_cEAP_Pep1Ch and SG200GRab5a_cEAP_ΔPit2_nPit2Ch that showed reduced or no EE motility. Images were taken at 200 ms exposure time. The average fluorescence intensity over length of ~5 μm was measured and corrected for the background intensity in the cytoplasm. All statistic testing was done using the software Prism 4.03 (GraphPad Software Inc.).

To analyse expression of cytoplasmic GFP expressed under the *cmu1* promoter, images (200 ms exposure time, spinning disc microscope) were taken of strains SG200Pcmu1-GFP, SG200ΔHok1_Pcmu1-GFP and SG200ΔCrk1_Pcmu1-GFP at 1 and 2 d.p.i. The average fluorescent intensity was measured and corrected for the background adjacent to the cell. All statistic testing was done using the software Prism 4.03 (GraphPad Software Inc.).

### Quantitative RT–PCR analysis

Quantitative analysis of effector expression was performed using qRT–PCR, according to published protocols^9^,^11^,^13^,^68^. In brief, leaf samples were removed 2–4 cm beneath the injection point and harvested from four independent experiments, 30 plants each. The material was frozen in liquid nitrogen, ground by mortar and pestle, and total RNA was extracted by using an RNeasy Plant Mini Kit (Qiagen, Hilden, Germany) with DNase I treatment, according to the manufacturer’s instructions. cDNA was synthesized using SuperScript III First-Strand Synthesis SuperMix (Invitrogen, Carlsbad, USA). qRT–PCR was performed with an Mx3005P thermocycler (Stratagene, La Jolla, USA) by using Brilliant III Ultra-Fast SYBR Green QPCR Master Mix (Stratagene) and ~250 or 500 ng cDNAs, estimated by NanoDrop ND-1000 (LabTech International Ltd, East Sussex, UK), from samples of liquid cultures or plants, respectively. qRT–PCR conditions were as follows: 3 min at 95 °C, followed by 40 cycles of 95 °C for 20 s and 60 °C for 20 s. To normalize expression levels, the constitutively expressed peptidyl–prolyl cis–trans isomerase gene (*ppi*) was used, as described previously^9^.

### Bioinformatics

Putative components of EE-associated signalling were identified by reciprocal BLAST search (http://blast.ncbi.nlm.nih.gov/Blast.cgi). All protein sequences used were fetched from the NCBI server (http://www.ncbi.nlm.nih.gov/pubmed). Accession codes of the protein sequences used in this approach were as follows: human ERK1: P27361.4; human ERK2: P28482.3; human MEK1: NP_005912.1; human MAPK p38: NP_002736.3; human Gab2: BAA76737.1; human RAP1: ABA64473.1; human GSK3beta: NP_002084.2; mouse RAF-B: NP_647455.3; human APPL1: NP_036228.1; human APPL2: NP_001238833.1; human SMAD2: AAC39657.1; human SMAD4: Q13485.1; human endofin: AAL30772.1 and human SARA: NP_004790.2.

## Author contributions

G.S. developed the research plan and the experimental strategy, directed the project, analysed the data, assembled all figures and movies, and wrote the manuscript (with the exception of Methods, which were mainly written by E.B., Y.H., M.S. and S.K.). E.B. generated 15 plasmids, 24 strains and designed the Δ*rab5a* experiments, analysed motor motility, diaminobenzidine precipitation and morphology of Δ*rab5a* and Δ*hok1* mutants. Y.H. generated 9 plasmids and 13 strains, partly performed and analysed the experiments of pathogenicity, effector transcription and secretion. M.S. analysed EE motility *in planta*, analysed dynein motility, Mcs1 secretion and effector secretion in Δ*hok1*, investigated co-motility of Crk1 and EEs, motility and nuclear localization of other signalling components, motility of Crk1 *in planta*, established the analysis of *P*_cmu1_-GFP reporter construct and analysed expression of this in control Δ*hok1* and Δ*crk1* mutants. S.K. generated four strains and one plasmid, participated in the scanning electron microscopy experiments and analysed expression of effectors in Δ*hok1* mutants. N.S. established, performed and analysed plant infection assays and diaminobenzidine staining of reactive oxygen. N.J.T. contributed to writing the manuscript and participated in the research plan design. All authors corrected the manuscript and discussed the data.

## Additional information

**How to cite this article:** Bielska, E. *et al*. Long-distance endosome trafficking drives fungal effector production during plant infection. *Nat. Commun.* 5:5097 doi: 10.1038/ncomms6097 (2014).

## Supplementary Material

Supplementary Information Supplementary Figures 1-6, Supplementary Tables 1-2, Supplementary Methods and Supplementary References

Supplementary Movie 1 Invading *U. maydis* hypha, 1 day after infection of a maize plant.

Supplementary Movie 2 Retrograde early endosome motility in an invading hyphal cell of *U. maydis* at 1 day after infection.

Supplementary Movie 3 Motility of early endosomes in the absence (no EAP) and presence (+EAP) of the endosome anchoring protein EAP.

Supplementary Movie 4 Motility of kinesin-3-GFP in a hyphal cell that expresses EAP.

Supplementary Movie 5 Endosome motility in invasive hyphal cells of *U. maydis* at various stages of pathogenic development.

Supplementary Movie 6 Endosome motility in invasive hyphal cells of *U. maydis* in the presence and absence of EAP expressed under the *crg1* or *mig1* promoter.

Supplementary Movie 7 Co-motility of Crk1 and early endosomes

Supplementary Movie 8 Motility of Crk1 in invading fungal hyphae at 1 dpi

## Figures and Tables

**Table 1:** Strains and plasmids used in this study.

**Strain or plasmid**	**Genotype**	**Reference**
SG200	*a1 mfa2 bW2 bE1, ble*^*R*^	35
SG200G_3__NLSR_3_	*a1 mfa2 bW2 bE1, ble*^*R*^/poG_3_/poNLS3RFP	This study
SG200Cmu1Ch_G_3_	*a1 mfa2 bW2 bE1*, P*cmu1-cmu1-mcherry, ble*^*R*^*, hyg*^*R*^/poG_3_	This study
SG200Cmu1Ch_paGRab5a_H4Ch	*a1 mfa2 bW2 bE1*, P*cmu1-cmu1-mcherry, ble*^*R*^*, hyg*^*R*^/pNopaGRab5a/pCoH4Ch	This study
AB33GRab5a	*a2* P*nar-bW2* P*nar-bE1*, *ble*^R^/poGRab5a	64
AB33GRab5a_cEAP	*a2* P*nar-bW2* P*nar-bE1, ble*^*R*^/poGRab5a/pHPcEAP	This study
AB33Kin3G_ChRab5a_cEAP	*a2* P*nar-bW2* P*nar-bE1*, P*kin3-kin3-egfp, ble*^*R*^, *hyg*^*R*^/po_m_ChRab5a/pCPcEAP	This study
SG200GRab5a	*a1 mfa2 bW2 bE1, ble*^*R*^/poGRab5a	This study
SG200GRab5a_cEAP	*a1 mfa2 bW2 bE1, ble*^*R*^/poGRab5a/pHPcEAP	This study
SG200GRab5a_mEAP	*a1 mfa2 bW2 bE1, ble*^*R*^/poGRab5a/pCPmEAP	This study
SG200GRab5a_cEAP_Cmu1Ch	*a1 mfa2 bW2 bE1*, P*cmu1-cmu1-mcherry, ble*^*R*^*, cbx*^*R*^/poGRab5a/pHPcEAP	This study
SG200GRab5a_cEAP_Pep1Ch	*a1 mfa2 bW2 bE1*, P*pep1-pep1-mcherry, ble*^*R*^*, cbx*^*R*^/poGRab5a/pHPcEAP	This study
SG200GRab5a_cEAP_ΔPit2_nPit2Ch	*a1 mfa2 bW2 bE1*, Δ*pit2, ble*^*R*^*, G418*^*R*^/poGRab5a/pCnPit2Ch	This study
SG200ΔKin3_GRab5a	*a1 mfa2 bW2 bE1*, Δ*kin3, ble*^*R*^*, nat*^*R*^/pCoGRab5a	This study
AB33ΔKin3_ChRab5a_Kin3G	*a2* P*nar-bW2* P*nar-bE1*, Δ*kin3, ble*^*R*^*, nat*^*R*^/pHo_m_ChRab5a/pKin3G	27
AB33G_3_Dyn2_ChRab5a	*a2* P*nar-bW2* P*nar-bE1*, P*dyn2-3xegfp-dyn2, ble*^*R*^*, hyg*^*R*^/po_m_ChRab5a	27
AB33G_3_Dyn2_ChRab5a_cEAP	*a2* P*nar-bW2* P*nar-bE1*, P*dyn2-3xegfp-dyn2, ble*^*R*^*, hyg*^*R*^/po_m_ChRab5a/pCPcEAP	This study
AB33Mcs1G_3_	*a2* P*nar-bW2* P*nar-bE1*, P*mcs1-mcs1-3xegfp, ble*^*R*^*, hyg*^*R*^	This study
AB33Mcs1G_3__cEAP	*a2* P*nar-bW2* P*nar-bE1*, P*mcs1-mcs1-3xegfp, ble*^*R*^*, hyg*^*R*^/pCPcEAP	This study
SG200*crg*G	*a1 mfa2 bW2 bE1, ble*^*R*^/pcrg1GFP	This study
SG200*mig*G	*a1 mfa2 bW2 bE1, ble*^*R*^/pmig1GFP	This study
SG200Cmu1Ch_G_3_	*a1 mfa2 bW2 bE1*, P*cmu1-cmu1-mcherry, ble*^*R*^*, hyg*^*R*^/poG_3_	This study
SG200GRab5a_Pep1Ch_G_3_	*a1 mfa2 bW2 bE1*, P*pep1-pep1-mcherry, ble*^*R*^*, hyg*^*R*^/poGRab5a/poG_3_	This study
SG200G_3__nPit2Ch	*a1 mfa2 bW2 bE1, ble*^*R*^/poG_3_/pHnPit2Ch	This study
SG200Cmu1Ch	*a1 mfa2 bW2 bE1*, P*cmu1-cmu1-mcherry, ble*^*R*^*, hyg*^*R*^	This study
AB33ΔHok1	*a2* P*nar-bW2* P*nar-bE1*, Δ*hok1, ble*^R,^, *hyg*^R^	37
AB33GRab5a_ΔHok1	*a2* P*nar-bW2* P*nar-bE1*, Δ*hok1, ble*^*R*^*, hyg*^*R*^*/* poGRab5a	37
SG200ΔHok1	*a1 mfa2 bW2 bE1*, Δ*hok1, ble*^*R*^*, hyg*^*R*^	This study
SG200nPit2Ch	*a1 mfa2 bW2 bE1, ble*^*R*^/pHnPit2Ch	This study
SG200nPit2Ch_ΔHok1	*a1 mfa2 bW2 bE1*, Δ*hok1, ble*^*R*^*, nat*^*R*^/pHnPit2Ch	This study
SG200Cmu1Ch_ΔHok1	*a1 mfa2 bW2 bE1*, P*cmu1-cmu1-mcherry*, Δ*hok1, ble*^*R*^*, hyg*^*R*^*, nat*^*R*^	This study
SG200Pep1Ch_ΔHok1	*a1 mfa2 bW2 bE1*, P*pep1-pep1-mcherry*, Δ*hok1, ble*^*R*^*, hyg*^*R*^*, nat*^*R*^	This study
AB33ΔRab5a	*a2* P*nar-bW2* P*nar-bE1*, Δ*rab5a, ble*^*R*^*, nat*^*R*^	This study
AB33ΔRab5a_Yup1G	*a2* P*nar-bW2* P*nar-bE1*, Δ*rab5a, ble*^*R*^*, nat*^*R*^/pYup1SG2	This study
SG200ΔRab5a	*a1 mfa2 bW2 bE1*, Δ*rab5a, ble*^*R*^*, nat*^*R*^	This study
SG200ΔRab5a_Cmu1Ch	*a1 mfa2 bW2 bE1*, Δ*rab5a*, P*cmu1-cmu1-mcherry, ble*^*R*^*, nat*^*R*^*, hyg*^*R*^	This study
SG200Pep1Ch	*a1 mfa2 bW2 bE1*, P*pep1-pep1-mcherry, ble*^*R*^*, hyg*^*R*^	This study
SG200ΔRab5a_Pep1Ch	*a1 mfa2 bW2 bE1*, Δ*rab5a*, P*pep1-pep1-mcherry, ble*^*R*^*, nat*^*R*^*, hyg*^*R*^	This study
SG200nPit2Ch_ΔRab5a	*a1 mfa2 bW2 bE1*, Δ*rab5a, ble*^*R*^*, nat*^*R*^/pHnPit2Ch	This study
AB33mChRab5a_Kpp2G	*a2* P*nar-bW2* P*nar-bE1*, *ble*^R^/po_m_ChRab5a/pKpp2G	This study
AB33mChRab5a_Kpp4G	*a2* P*nar-bW2* P*nar-bE1*, *ble*^R^/po_m_ChRab5a/pKpp4G	This study
AB33mChRab5a_Fuz7G	*a2* P*nar-bW2* P*nar-bE1*, *ble*^R^/po_m_ChRab5a/pFuz7G	This study
AB33mChRab5a_Gsk3βG	*a2* P*nar-bW2* P*nar-bE1*, *ble*^R^/po_m_ChRab5a/pGsk3βG	This study
AB33mChRab5a_Kpp6G	*a2* P*nar-bW2* P*nar-bE1*, *ble*^R^/po_m_ChRab5a/pKpp6G	This study
AB33mChRab5a_Crk1G	*a2* P*nar-bW2* P*nar-bE1*, *ble*^R^/po_m_ChRab5a/pCrk1G	This study
AB33mChRab5a_Crk1G_3_	*a2* P*nar-bW2* P*nar-bE1*, P*crk1-crk1-3xegfp, ble*^R^*, hyg*^*R*^/po_m_ChRab5a	This study
SG200Crk1G_3_	*a1 mfa2 bW2 bE1*, P*crk1-crk1-3xegfp, ble*^R^*, hyg*^*R*^	This study
SG200*cmu1*G	*a1 mfa2 bW2 bE1, ble*^*R*^*/*pcmu1GFP	This study
SG200Cmu1Ch_ΔCrk1	*a1 mfa2 bW2 bE1*, P*cmu1-cmu1-mcherry*, Δ*crk1, ble*^*R*^*, hyg*^*R*^, *G418*^*R*^	This study
SG200*cmu1*G_ΔCrk1	*a1 mfa2 bW2 bE1*, Δ*crk1, ble*^*R*^*, G418*^*R*^*/*pcmu1GFP	This study
SG200*cmu1*G_ΔHok1	*a1 mfa2 bW2 bE1*, Δ*hok1, ble*^*R*^*, hyg*^*R*^*/*pcmu1GFP	This study
poG_3_	P*otef-3xegfp, cbx*^*R*^	This study
poNLS3RFP	P*otef-gal4s-3xmrfp, nat*^*R*^	27
pNopaGRab5a	P*otef-pagfp-rab5a, nat*^*R*^	This study
pCoH4Ch	P*otef-his4-mcherry, cbx*^*R*^	This study
pHCmu1Ch	P*cmu1-cmu1-mcherry, hyg*^*R*^	This study
pCCmu1Ch	P*cmu1-cmu1-mcherry, cbx*^*R*^	This study
pHPep1Ch	P*pep1-pep1-mcherry, hyg*^*R*^	This study
pCPep1Ch	P*pep1-pep1-mcherry, cbx*^*R*^	This study
poGRab5a	P*otef-egfp-rab5a, nat*^*R*^	64
pCoGRab5a	P*otef-egfp-rab5a, cbx*^*R*^	65
po_m_ChRab5a	P*otef-mcherry-rab5a, nat*^*R*^	27
pHo_m_ChRab5a	P*otef-mcherry-rab5a, hyg*^*R*^	33
pKin3G	P*kin3-kin3-egfp, cbx*^*R*^	69
pHPcEAP	P*crg1-kin1*^*G96E,1-739*^*-PX, hyg*^*R*^	This study
pCPcEAP	P*crg1-kin1*^*G96E,1-739*^*-PX, cbx*^*R*^	This study
pCPmEAP	P*mig1-kin1*^*G96E,1-739*^*-PX, cbx*^*R*^	This study
pΔPit2	Δ*pit2, G418*^*R*^	This study
pCnPit2Ch	P*pit2-pit2-mcherry, cbx*^*R*^	This study
pHnPit2Ch	P*pit2-pit2-mcherry, hyg*^*R*^	This study
pYup1SG2	P*otef-yup1-sgfp, cbx*^*R*^	23
pMcs1G_3_	P*mcs1-mcs1-3xegfp, hyg*^*R*^	This study
p^H^ΔHok1	Δ*hok1, hyg*^*R*^,	37
p^N^ΔHok1	Δ*hok1, nat*^*R*^	37
pΔRab5a	Δ*rab5a, nat*^*R*^	This study
pcrg1GFP	P*crg1-egfp, cbx*^*R*^	This study
pmig1GFP	P*mig1-egfp, cbx*^*R*^	This study
pKpp2G	P*otef-kpp2-egfp, cbx*^*R*^	This study
pKpp4G	P*otef-kpp4-egfp, cbx*^*R*^	This study
pFuz7G	P*otef-fuz7-egfp, cbx*^*R*^	This study
pGsk3βG	P*otef-gsk3*β*-egfp, cbx*^*R*^	This study
pKpp6G	P*otef-kpp6-egfp, cbx*^*R*^	This study
pCrk1G	P*otef-crk1-egfp, cbx*^*R*^	This study
pCrk1G_3_	P*crk1-crk1-3xegfp, hyg*^*R*^	This study
pΔCrk1	Δ*crk1, G418*^*R*^	This study
pcmu1GFP	P*cmu1-egfp, cbx*^*R*^	This study

Δ, deletion; ‘−’, fusion; ‘/’, ectopically integrated; *a*, *b*, mating type loci; *ble*^*R*^, phleomycin resistance; *cbx*^*R*^, carboxin resistance; *cmu1*, chorismate mutase; *crg1*, conditional arabinose-induced promoter; *dyn2*, C-terminal half of the dynein heavy chain; EAP, synthetic early endosomes anchoring protein; *E1*, *W2*, genes of the *b* mating type loci; *egfp*, enhanced green fluorescent protein; *G418*^*R*^, geneticin resistance; *his4*, histone-4; *hyg*^*R*^, hygromycin resistance; *kin1*, kinesin-1; *kin3*, kinesin-3; *mcherry*, monomeric cherry; *mcs1*, myosin-chitin synthase 1; *mig1*, maize-inducible promoter; *mrfp*, monomeric red fluorescent protein; *nat*^*R*^, nourseothricin resistance; NLS, nuclear localization signal of the GAL-4 DNA-binding domain from pC-ACT1 (Clontech, Mountain View, CA, USA); *otef*, constitutive promoter; P, promoter; *pagfp*, photo-activatable monomeric green fluorescent protein; *pep1*, immunity suppressor; *pit2*, protease inhibitor; *PX*, Phox domain from Yup1 (aa 4-148); *rab5a*, small endosomal Rab5-like GTPase; *yup1*, endosomal t-SNARE.
