# Supplementary material for: Long-distance endosome trafficking drives fungal effector production during plant infection
Source: Nat Commun. 2014 Oct 6;5:5097. doi: 10.1038/ncomms6097 (PMC4205857; doi:10.1038/ncomms6097)
Supplement: Supplementary Information — Supplementary Figures 1-6, Supplementary Tables 1-2, Supplementary Methods and Supplementary References [file ncomms6097-s1.pdf]

Supplementary Figures and Legends

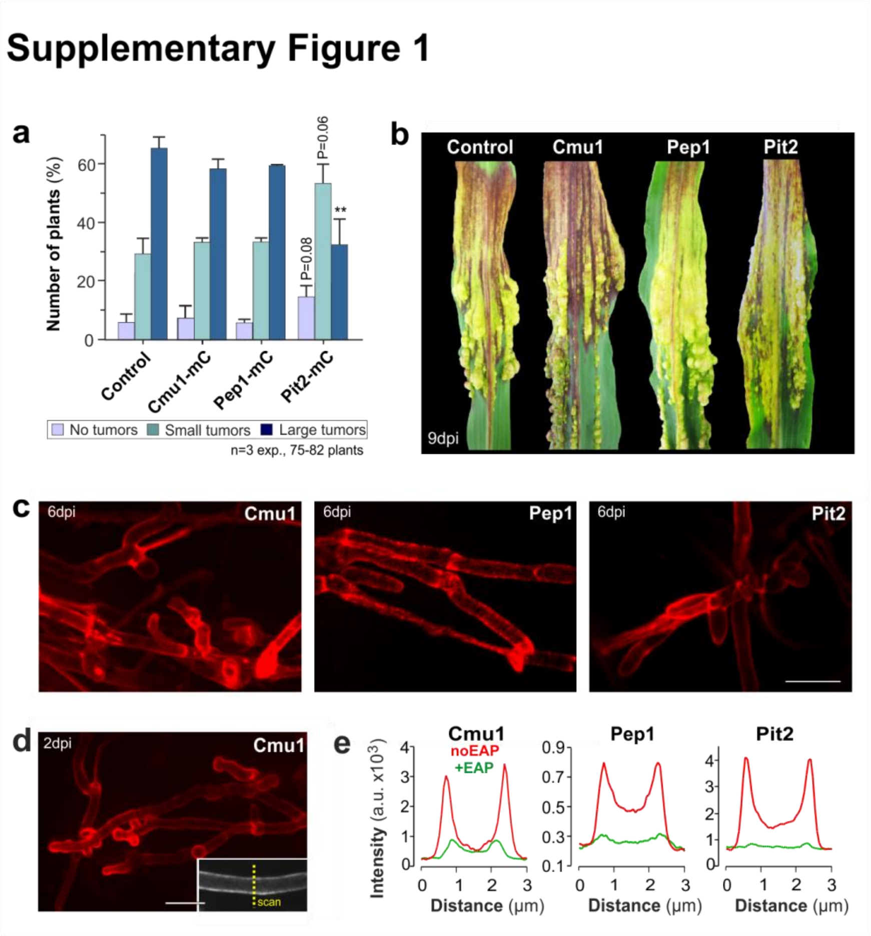

Supplementary Figure 1. Virulence of *U. maydis* strains expressing fluorescently-labelled effector proteins.

a, Plant disease symptoms in Control, and mutants expressing mCherry-labelled Cmu1 (Cmu1-mC), Pep1 (Pep1-mC) and Pit2 (Pit2-mC). Mean ± SE and sample size *n* is shown. Borderline significance P values are given; **= significance at *P*=0.0014; unpaired Student t-test.

b, Maize leaves infected with control strains and strains expressing mCherry-labelled Cmu1 (Cmu1), Pep1 (Pep1) and Pit2 (Pit2) at 9 dpi.

c, Hyphal cells during early infection showing apical secretion of Cmu1-mCherry (Cmu1, red), Pep1-mCherry (Pep1, red) and Pit2-mCherry (Pit2, red). Scale bar, 10 µm.

d, Secretion of Cmu1-mCherry in invasive hyphae at 2 dpi. Lower inset shows scanning area used in Supplementary Fig. 1e. Scale bar, 10 µm.

e, Fluorescent intensity profiles of Cmu1-mCherry, Pep1-mCherry and Pit2-mCherry in invasive hyphae in plants at 2 dpi. In the absence of EAP (no EAP, red), effectors concentrate at the cell edge; inhibition of EE motility in the presence of EAP (+EAP, green) reduces the fluorescent signals. Scan lines are indicated in Supplementary Fig.1d, lower inset.

Supplementary Figure 2

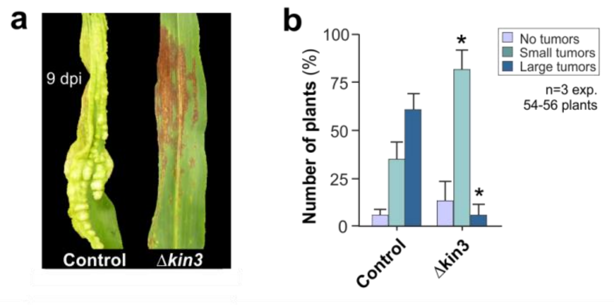

Supplementary Figure 2. Plant infection by *kin3* null mutants.

a, Control strains induce large tumors and abnormal plant growth (Control), whereas Δ*kin3* mutants cause minor symptoms (Δ*kin3*). Plants are shown at 9 days after infection.

b, Plant symptoms at 14 days after infection with control and Δ*kin3* deletion mutants. Means ± SE and sample sizes *n* (= number of experiments) are shown. *= significance at *P*=0.0390 (small tumors) and *P*=0.0137 (large tumors), unpaired Student t-test.

Supplementary Figure 3

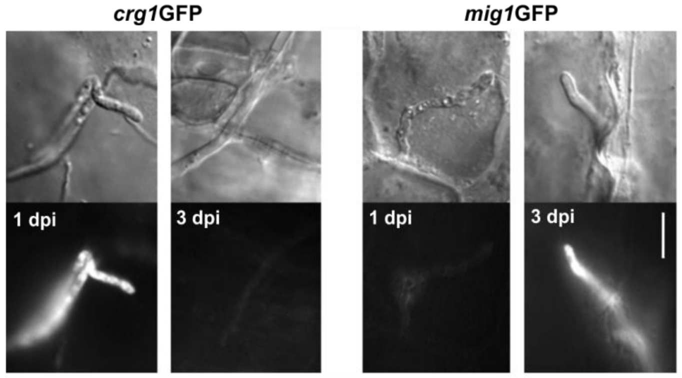

Supplementary Figure 3. Expression of cytoplasmic GFP under control of the *crg1* or the *mig1* promoter in invasive hyphae of *U. maydis*.

When GFP is expressed under the control of arabinose-inducible *crg1*-promoter, strong cytoplasmic fluorescence is seen at 1 dpi, but no fluorescence was detected at 3 dpi (*crg1*GFP). In contrast in cells expressing GFP under the plant-induced *mig1* promoter (*mig1*GFP), fluorescence is absent at early stages (1 dpi), but appears after several days of plant colonization (3 dpi). Scale bar, 6 µm.

Supplementary Figure 4

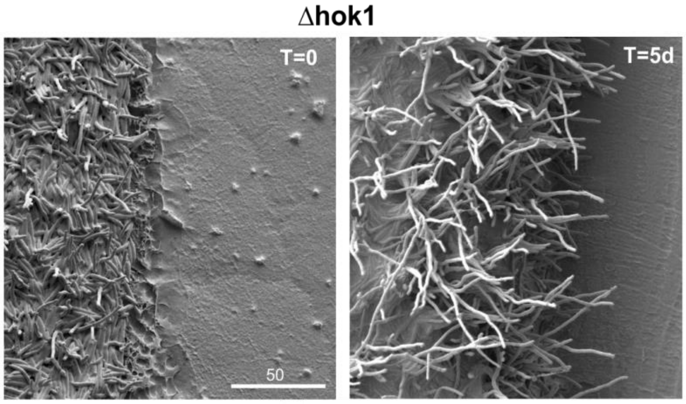

Supplementary Figure 4. Induction of hyphal growth in strain SG200ΔHok1.

Cells of strain SG200ΔHok1 are deleted in the motor adapter Hok1 and show strongly impaired EE motility[4]. Nevertheless, they switch from yeast-like cells to hyphae after 5 days (T=5) on charcoal-containing agar. This indicates that pheromone-based auto-stimulation and *b*-dependent filamentous growth occurs in the absence of EE motility. Scale bar, 50 µm.

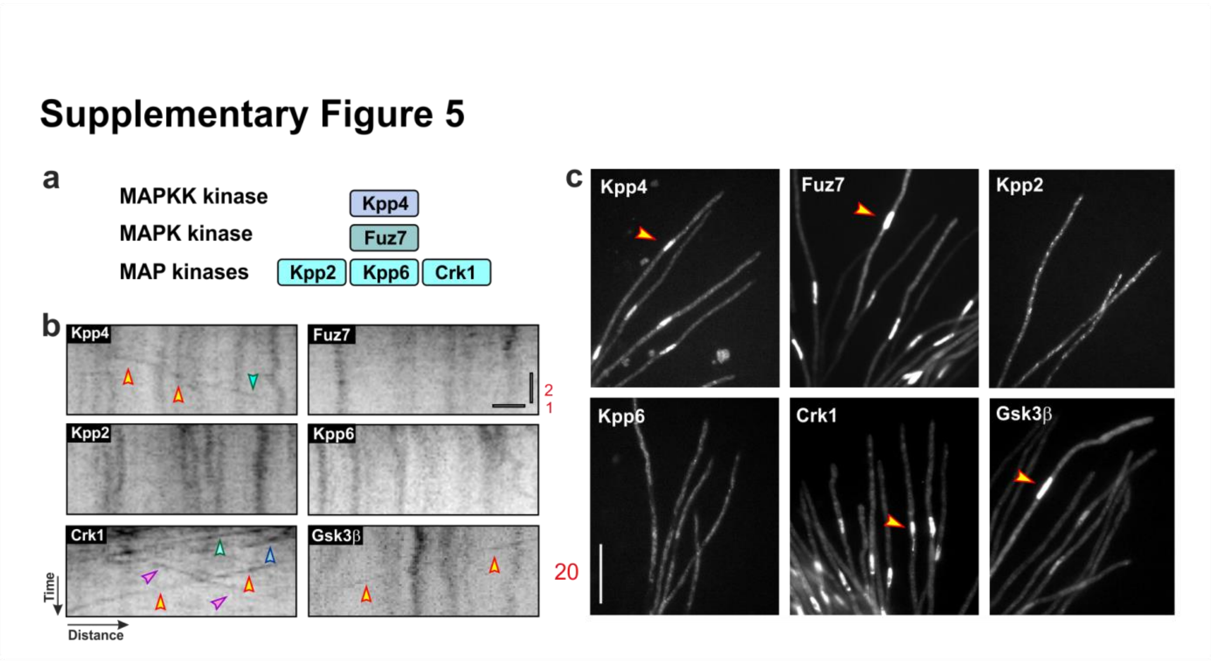

Supplementary Figure 5. Live imaging to show MAP kinases motility in *U. maydis.*

a, MAPK module components in *U. maydis*.

b, Contrast inverted kymographs showing motility of MAPK module components and a homologue of the human glycogen synthase kinase-3 beta (Gsk3β). MAPK Crk1 shows prominent directed movements and the MAPKK kinase Kpp4 and Gsk3β occasionally move (individual trajectories are highlighted by coloured arrowheads). The MAPK kinase Fuz7, and the MAPKs Kpp2 and Kpp6 are immobile. Scale bars, 2 s (vertical) and 1 µm (horizontal).

c, Cellular localisation of MAPK module components and a Gsk3β homologue. With the exception of Kpp2 and Kpp6, all proteins localize to the nucleus (arrowhead). Scale bar, 20 µm.

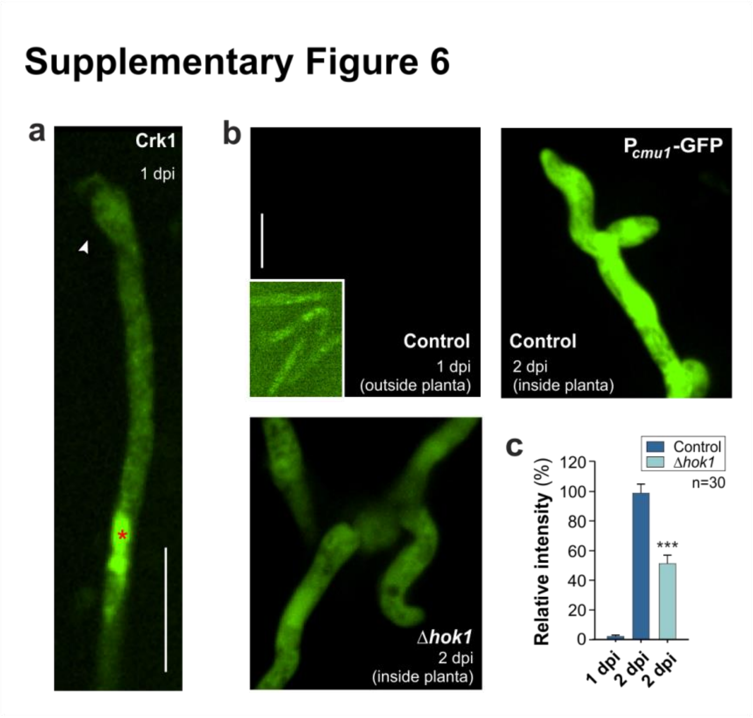

Supplementary Figure 6. Induction of the *cmu1* promoter *in planta* in control cells and *hok1* null mutants.

a, Images showing Crk1-GFP_3 in an invasive hypha on top of a plant leaf. The appressorium is marked by arrowhead. Note that the MAPK accumulates in the nucleus (indicated by asterisk). Scale bar is 10 µm.

b, Control cell on the surface of a plant leave. The cell contains cytoplasmic GFP under the control of the *cmu1* promoter. At 1 dpi, the effector promoter is not yet induced and, consequently, no fluorescence is visible (inset provides same field of observation at different image scaling to show presence of cells). Upon invasion, the *cmu1* promoter is induced and cytoplasmic GFP is filling the cell (Control, 2 dpi). In *hok1* null mutants, EE motility is strongly impaired and expression from the *cmu1* promoter is reduced (Δ*hok1*). Images ware scaled identically to show different degrees cytoplasmic GFP expression. Scale bar, 5 µm.

c, Expression of cytoplasmic GFP under the control of *cmu1* promoter in invasive control and Δ*hok1*mutant hyphae in plants. Mean ± SE and sample size *n* from two experiments is shown. ***= significance at error probability P<0.0001; unpaired Student t-test.

Supplementary Tables

Supplementary Table 1. Experimental usage of strains

Strain	Experiment	Figure or Video
SG200	Hyphal image and pathogenicity assay	Fig. 1a, 5(c, d and f), 6(c, d and f)
SG200_G_3_$NLSR_3$	Hyphal penetration image with nucleus	Fig. 1b, Supp. Movie 1
SG200Cmu1Ch_G_3	Cmu1-mCherry secretion image	Fig. 1c, Supp. Fig. 1c
SG200Cmu1Ch_paGRab5a_H4Ch	EE motility analysis	Fig. 1(d and e), Supp. Movie 2
SG200GRab5a	EE motility analysis and pathogenicity assay	Fig. 2c, 3, , Supp. Fig. 2, Supp. Movie 5
SG200ΔKin3_GRab5a	Pathogenicity assay	Supp. Fig. 2
AB33GRab5a_cEAP	EE motility analysis +/no EAP	Fig. 2(b and c), Supp. Movie 3
AB33GRab5a	EE motility analysis in hyphae	Fig. 2c
AB33Kin3G_ChRab5a_cEAP	Analysis of kinesin-3-GFP independent of EEs	Fig. 2(d and e), Supp. Movie 4
AB33ΔKin3_ChRab5a_Kin3G	Motility analysis of kinesin-3-GFP independent of EEs	Fig. 2e
SG200GRab5a_cEAP	Analysis of EE motility, pathogenicity and effectors expression with EAP under *crg1* promoter	Fig. 2c, 3(b-g), 4c, d, , Supp. Fig. 1(a and b), Supp. Movie 6
AB33G_3Dyn2_ChRab5a_cEAP	Dynein and EE motility analysis with EAP under *crg1* promoter	Fig. 2(f-h)
AB33Mcs1G_3	Mcs1 secretion analysis	Fig. 2(i and j)
AB33Mcs1G_3_cEAP	Mcs1 secretion analysis with EAP under *crg1* promoter	Fig. 2(i and j)
SG200*crg1*G	Cytoplasmic GFP expression analysis under *crg1* promoter	Supp. Fig. 3
SG200*mig1*G	Cytoplasmic GFP expression analysis under *mig1* promoter	Supp. Fig. 3
SG200GRab5a_mEAP	Analysis of EE motility and pathogenicity with EAP under *mig1* promoter	Fig. 3(b-g), Supp. Movie 6
SG200GRab5a_cEAP_Cmu1Ch	Cmu1-mCherry secretion analysis and pathogenicity assay with EAP under *crg1* promoter	Fig. 4(a and b), Supp. Fig. 1(a, b and e)
SG200GRab5a_cEAP_Pep1Ch	Pep1-mCherry secretion and pathogenicity assay with EAP under *crg1* promoter	Fig. 4(a and b), Supp. Fig. 1(a, b and e)
SG200GRab5a_cEAP_ΔPit2_nPit2Ch	Pit2-mCherry secretion and pathogenicity assay with EAP under *crg1* promoter	Fig. 4(a and b), Supp. Fig. 1(a, b and e)
SG200GRab5a_Pep1Ch_G_3	Pep1-mCherry secretion analysis	Supp. Fig. 1c
SG200G_3_nPit2Ch	Pit2-mCherry secretion analysis	Supp. Fig. 1c
SG200Cmu1Ch	Cmu1-mCherry secretion analysis	Fig. 5(e and g), 6(e and g), 7(c and d), Supp. Fig. 1d
AB33ΔHok1	Phenotypic analysis of ΔHok1strain	Fig. 5a
AB33GRab5a_ΔHok	EE motility analysis of ΔHok1 strain	Fig. 5b
SG200ΔHok1	Analysis of pathogenicity and effectors expression	Fig. 5(c, d and f), Supp. Fig. 4
SG200nPit2Ch_ΔHok1	Pit2-mCherry secretion analysis in ΔHok1 strain	Fig. 5e
SG200Cmu1Ch_ΔHok1	Cmu1-mCherry secretion analysis in ΔHok1 strain	Fig. 5(e and g)
SG200Pep1Ch_ΔHok1	Pep1-mCherry secretion analysis in ΔHok1 strain	Fig. 5e
AB33ΔRab5a	Phenotypic analysis of ΔRab5a strain	Fig. 6a
AB33ΔRab5a_Yup1G	EE motility analysis of ΔRab5a strain	Fig. 6b
SG200ΔRab5a	Analysis of pathogenicity and effectors expression	Fig. 6(c, d and f)
SG200ΔRab5a_Cmu1Ch	Cmu1-mCherry secretion analysis in ΔRab5a strain	Fig. 6(e and g)
SG200Pep1Ch	Pep1-mCherry secretion analysis	Fig. 6e
SG200ΔRab5a_Pep1Ch	Pep1-mCherry secretion analysis in ΔRab5a strain	Fig. 6e
SG200nPit2Ch	Pit2-mCherry secretion analysis	Fig. 6e
SG200nPit2Ch_ΔRab5a	Pit2-mCherry secretion analysis in ΔRab5a strain	Fig. 6e
AB33mChRab5a_Kpp2G	Cellular localisation of MAP kinase module components	Supp. Fig. 5(b and c)

AB33mChRab5a_Kpp4G	Cellular localisation of MAP kinase module components	Supp. Fig. 5(b and c)
AB33mChRab5a_Fuz7G	Cellular localisation of MAP kinase module components	Supp. Fig. 5(b and c)
AB33_mChRab5a_Gsk3βG	Cellular localisation of MAP kinase module components	Supp. Fig. 5(b and c)
AB33mChRab5a_Kpp6G	Cellular localisation of MAP kinase module components	Supp. Fig. 5(b and c)
AB33mChRab5a_Crk1G	Cellular localisation of MAP kinase module components	Supp. Fig. 5(b and c)
AB33mChRab5a_Crk1G_3	Cellular localisation of MAP kinase module components	Fig. 7a, Supp. Movie 7
SG200Crk1G_3	Motility analysis of Crk1G_3 in infectious hyphae	Fig. 7b, Supp. Movie 8
SG200Cmu1Ch_ΔCrk1	Cmu1-mCherry secretion analysis in ΔCrk1 strain	Fig. 7(c and d)
SG200*cmu1*G	Cytoplasmic GFP expression analysis under *cmu1* promoter	Fig. 7(e and f), Supp. Fig. 6
SG200*cmu1G*_ΔCrk1	Cytoplasmic GFP expression analysis under *cmu1* promoter in Δ*crk1* null mutant	Fig. 7(e and f)
SG200*cmu1*G_ΔHok1	Cytoplasmic GFP expression analysis under *cmu1* promoter in Δ*hok1* null mutant	Supp. Fig. 6

Supplementary Table 2. Primers used in this study

Primer	Sequence 5' to 3'
EB170	GGTGAAACTCGATGAGGCCAAAAAAGATACATGTCCAACAACATCAAGGTC
EB171	AGTGGTGACGGCAAACGCGCG
EB172	GAGCTCAAGCGCGCGTTTGCCGTCACCACTACACAGCCACTCCAAGGAATC
EB173	GAACGATCTGCAGCCGGGCGGCCGCTTTACGGCCATTCGATAAACTGCTTG
EB192	CCATCAACGTGCTCGATGCGGCCGCGGCGCTACGTAGAGAGGCGTGATCTC
EB193	GAAAACGACAGACGACCTTGATGTTGTTGGACATCTTGATCTGGAGGAAGAGAATG
EB277	GCAAGACCGGCAACAGGATTC
EB298	GCTTCCGGCTCGTATGTTGTG
EB306	GGTGAAACTCGATGAGGCCAAAAAAGATACATGGTGAGCAAGGGCGAGGAG
EB307	CTTAGTATTCCATTCTCTTCCTCCAGATCAAGATGGTGAGCAAGGGCGAGGAG
EB308	CTTGATCTGGAGGAAGAGAATG
EB310	GACCTGCCCCAAGAACCTCAAC
EB475	GCCGGCAGATCCGATGTACC
EB476	CACTCCTACAGCTTGCAAATTAAAG
EB513	CCAAAAAAGAAGAGAAAGGTCGAATTCCATATGTCACATGCCCACGGACAG
EB514	GGTGAACAGCTCCTCGCCCTTGCTCACCATACGCATGATCTCGTTATAAATCAAC
EB515	CCAAAAAAGAAGAGAAAGGTCGAATTCCATATGGGCACAGGCGTCATGGCC
EB516	GGTGAACAGCTCCTCGCCCTTGCTCACCATAGAATCCGCCTCTTCGCTGAAC
EB517	CCAAAAAAGAAGAGAAAGGTCGAATTCCATATGCTTTCGTCCGGTGCGGG
EB518	GGTGAACAGCTCCTCGCCCTTGCTCACCATCTTCATCCCATCGGCCCATG
EB519	CCAAAAAAGAAGAGAAAGGTCGAATTCCATATGTCTAACGCGCCGCTCAAC
EB520	GGTGAACAGCTCCTCGCCCTTGCTCACCATGTCAAGCGTGACCTTCAGCG
EB521	CCAAAAAAGAAGAGAAAGGTCGAATTCCATATGTCGATTGCCAATGCCTCTTC
EB522	GGTGAACAGCTCCTCGCCCTTGCTCACCATACGAAGAAGCGGCTGAAATTCTTG
EB523	CCAAAAAAGAAGAGAAAGGTCGAATTCCATATGGCACAGGTTGCTTCCAGC
EB524	GGTGAACAGCTCCTCGCCCTTGCTCACCATGCTTTGCGGCAATGGACCGG

EB543	GATGCGGCCAGCAAAACTAAAAAACTGTATTATAAGCCATCCATGCCCAGCTTTGC
EB544	GGTGAACAGCTCCTCGCCCTTGCTCACCATGCTTTGCGGCAATGGACCGG
EB545	GCTTGGCACTGGCCGTCGTTTTACAACGTCTCATCGATGATCTCGCATTCTAC
EB546	GTGTGGAATTGTGAGCGGATAACAAGGATCCGATAGAAGATGAGTGGGGCGC
EB580	GAAAAACTGTTGGGAAGGGCGATCGGTGCGGGCCGAATTCCACACTTTGAGCTGCCGCACG
EB581	GCTCGGTACGGGTACATCGGATCTGCCGGCGCGGCCGCAGCTTAGATGGTGATGGTTCGC
EB582	CGAAGGCTTTAATTTGCAAGCTGTAGGAGTGGCGGCCGCTCATCGATGATCTCGCATTC
EB583	GTATGTTGTGTGGAATTGTGAGCGGATAACAAGAATTCCAAGATAGAAGATGAGTGGGG
EB700	GAAAAACTGTTGGGAAGGGCGATCGGTGCGGGCCCAGATTCACATTTCGCCTCACG
EB701	GGTGAACAGCTCCTCGCCCTTGCTCACCATCGTAACCTAGAGCTCTTGCAG
ODA148	GCACGGTGATCACGCCT
ODA149	CACGGCCTGAGTGGCCGACCAGACGCTTTTCGGG
ODA150	GTGGGCCATCTAGGCCCAGGCGGAACTGCATCTC
ODA151	CGGCTGACTTAGAACGCG
OWI114	GCCATCCATATGCGGTGCGTTCGCTCCTGG
OWI116	GATACGCGTGTGCGTGTGTTTG
OWI117	TTAAGCTTCGTACCACGTCACTAACCC
OWI120	GCTCACCATGGCGCCCGACTCGGAGAG
YH166	ATGGTGAGCAAGGGCGAGGAG
YH352	AACTGTTGGGAAGGGCGATCGGTGCGGGCCTGTGGCTCACAAACGTCTGTG
YH353	CATGTTATCCTCCTCGCCCTTGCTCACCATCATGCCAAACATGCTACCGATTC
YH354	CCGGCTGTGCTTCAATCGCTGCTGCGCCGCACCGCTGCGACGTCGTTGATG
YH355	ATGTTGTGTGGAATTGTGAGCGGATAACAAGCCATGCTTTTTCACGCTAGAACTCAC
YH360	AACTGTTGGGAAGGGCGATCGGTGCGGGCCCGAGCGTGGAATGCTGGCATG
YH361	CATGTTATCCTCCTCGCCCTTGCTCACCATGGTGCACTTGTTGGCGTGGTC
YH362	CCGGCTGTGCTTCAATCGCTGCTGCGCCGCGCCAACCCCTCATCCACTTGC
YH363	ATGTTGTGTGGAATTGTGAGCGGATAACAAGGTGGTGGAAGACGATCCGATC
YH372	CAGAGTCATCATCAACGACGTCGCAGCGGTCATCGAGCACGTTGATGGGATCGATCC
YH373	AAGGGGTGTGCAAGTGGATGAGGGGTTGGCCATCGAGCACGTTGATGGGATCGATCC
YH397*	GAGCCTGCTCCATCTCGTCT
YH398*	GGATGTGGCAGAAGTTGTCG
YH399*	ACATCGTCAAGGCTATCG
YH400*	AAAGAACACCGGACTTGG
YH401*	CACTGACGACGACACCT
YH402*	TGCTACCGATTCCTCCT
YH403*	CAAGAATCCGCCTGCCAAC
YH404*	AGGATCTGTCGGCATGAC
YH518	AACTGTTGGGAAGGGCGATCGGTGCGGGCCCGCCGTCGCAAGAAACTTGTTGC
YH519	GCTCGGTACGGGTACATCGGATCTGCCGGCAGCGATCGACGCACCGGAATC
YH520	GAAGGCTTTAATTTGCAAGCTGTAGGAGTGGGTCATGCCGACAGATCCTAAC
YH521	ATGTTGTGTGGAATTGTGAGCGGATAACAAGAATTCCGCGGCTTCATCTTCGATG
YH527	GCCGGCAGATCCGATGTACCC
YH528	CACTCCTACAGCTTGCAAATTAAAGCCTTCG
YH539	AACTGTTGGGAAGGGCGATCGGTGCGGGCCTCACATGTATGGGGCCGGAAG
YH540	CATGTTATCCTCCTCGCCCTTGCTCACCATGGCGGTGGCGATCGAGCGTTCCCAGATGACCACATCTCCG
YH69	TAGAAAACAAAATATAGCGCGCCGCGGCCGGGCCGCGGGGATCTTCGCTC

*primers were previously published (ref. 12, 15, 16)

Supplementary Methods

Plasmid cloning. All plasmids were generated by standard cloning procedures or *in vivo* recombination in the yeast *Saccharomyces cerevisiae*[1]. Their genotype is summarised in Table 1 and their usage is indicated in Supplementary Table 1. Cloning primers are provided in Supplementary Table 2. Plasmids were generated as follows:

pHCmu1Ch and pHPep1Ch**.** The plasmids were used to visualize Cmu1-mCherry or Pep1-mCherry in plants. Both genes are expressed under their native promoters. An *S. cerevisiae* - *E. coli* shuttle vector sequence of 2680 bp fragment, containing the *S. cerevisiae URA3* marker, 2 µm *ori*, the ampicillin resistance cassette, an *E. coli* origin of replication and a 3735 bp fragment, containing *mCherry* and the hygromycin resistance cassette (hyg^R) was amplified by polymerase chain reaction (PCR). 1019 bp and 1000 bp fragments, including the entire *cmu1* and *pep1*, but excluding the stop codon, and 1011 bp and 484 bp of downstream of *cmu1* and *pep1*, respectively, were amplified by PCR using primers YH352, YH353, YH354, YH355, YH360, YH361, YH362 and YH363 (see Supplementary Table 2), generating a 30 bp overlap upstream and downstream of the vectors. pHCmu1Ch was generated by *in vivo* recombination[1], digested with *Psi*I, *Sph*I and *SnaB*I and a 5989 bp fragment was integrated into the *cmu1* locus of strains SG200, SG200GRab5a, SG200ΔRab5a and SG200GRab5a_mEAP, resulting in SG200Cmu1Ch, SG200GRab5a_Cmu1Ch, SG200ΔRab5a_Cmu1Ch and SG200GRab5a_mEAP_Cmu1Ch, respectively. For *U. maydis* transformation, pHPep1Ch was digested with *Hpa*I and *Psi*I. A 5136 bp fragment was integrated into

the *pep1* locus of strains SG200 and SG200GRab5a, resulting in SG200Pep1Ch and SG200GRab5a_Pep1Ch.

***pCCmu1Ch* and *pCPep1Ch*.** These plasmids were used to visualize secretion of Cmu1-mCherry or Pep1-mCherry. Both constructs are expressed under their native promoters. To replace hyg^R with carboxin gene resistance cassette (cbx^R), either pHCmu1Ch or pHPep1Ch was digested with both *Eco*RI and *Xba*I, and cbx^R was amplified by PCR using primers YH 69, YH372 and YH373 with 30 bp homology sequences of the vector for pCCmu1Ch or pCPep1Ch, respectively. The plasmid pCCmu1Ch was digested with *Psi*I, *Sph*I and *Sna*BI and a 4835 bp fragment was integrated into the *cmu1* locus of the strain SG200GRab5a_cEAP, resulting in SG200GRab5a_cEAP_Cmu1Ch. The plasmid pCPep1Ch was digested with *Psi*I and *Hpa*I and a 4285 bp fragment was integrated into the *pep1* locus of the strain SG200GRab5a_cEAP, resulting in SG200GRab5a_cEAP_Pep1Ch.

***poG$_3$*.** This plasmid contains 3 copies of *egfp* encoding region fused behind the *otef* promoter and cbx^R. Two additional copies of *egfp* were introduced into *Bsr*GI site of *egfp* in p123 (ref. 2).

pNopaGRab5a. This plasmid contains a *pagfp-rab5a* encoding region fused behind the *otef* promoter and nourseothricin resistance cassette (nat^R). For visualization of EEs after photoactivation, cbx^R of popaGRab5a (ref. 3) was replaced with nat^R and the plasmid was integrated into SG200Cmu1Ch resulting in SG200Cmu1Ch_paRab5a.

pCoH4Ch. This plasmid contains a fusion of the histone 4 gene *his4* and *mCherry*, which is placed under the control of the *otef* promoter and cbx^R. To visualize the

nucleus, plasmid pCoH4Ch was generated by exchanging *rfp* in the plasmid pCoH4R. The plasmid pCoH4R was digested with *Nco*I and *Eco*RV to obtain a 5683 bp fragment containing *otef* promoter, *his4*, the *Tnos* terminator and cbx^R. The fragment was ligated to a 742 bp *Nco*I-*Eco*RV fragment of *mCherry* resulting in the plasmid pCoH4Ch.

***pcrg1GFP*.** This plasmid contains an *egfp* encoding region fused behind the *crg1* promoter. A 827 bp fragment, encoding *egfp* and a fragment of the *Tnos* terminator (using primers EB306 and EB277) was cloned into the plasmid pcrgPeb1$^{211–268}$ (ref. 3), linearized with *EcoN*I and *Afl*II. The resulting plasmid pcrg1GFP was digested with *Age*I and integrated into the succinate dehydrogenase locus of the SG200 strain resulting in SG200*crg*G.

pmig1GFP. This plasmid contains an *egfp* encoding region fused behind the *mig1* promoter. A 1049 bp fragment of the *mig1* promoter and a 30 bp overhang was amplified from the plasmid pCPmEAP using primers EB192 and EB308. A second 1135 bp fragment, encoding *egfp* and *Tnos* terminator, was amplified from plasmid pcrg1GFP using primers EB307 and EB298. The fragments were cloned into the linearized (*BamH*I and *Sph*I) yeast-*E.coli* vector pNEBcbx-yeast-SspI (ref. 4) by yeast recombination. The resulting pmig1GFP was linearized and integrated into the succinate dehydrogenase locus of the SG200, resulting in SG200*mig*G. The presence of the *egfp* gene under *mig1* promoter was confirmed by PCR.

pcmu1GFP. This plasmid contains an *egfp* encoding region fused behind the *cmu1* promoter. A 1001 bp fragment of the *cmu1* promoter containing 30 bp overhangs was amplified from genomic DNA using primers EB700 and EB701. A second 1411 bp

fragment, encoding *egfp*, *Tnos* terminator and a fragment of the cbx^R, was amplified from the plasmid p^CHok1G (ref. 4) using primers YH166 and EB310. The fragments were cloned into the linearized (*EcoR*I and *Sac*I) yeast-*E.coli* vector pNEBcbx-yeast-SspI (ref. 4) by yeast recombination. The resulting pcmu1GFP was linearized with *Ssp*I and integrated into the succinate dehydrogenase locus of the SG200, resulting in SG200*cmu*G. The presence of the *egfp* gene under the control of *cmu1* promoter was confirmed by PCR.

***HPcEAP*, *pCPcEAP*, *pCPmEAP*.** These plasmids contain a *kin1* ^{rigor} (G96E) motor head, fused to the central coiled-coil[5], which is fused to the Phox domain from the putative t-SNARE Yup1 (ref. 6). This construct was placed behind the *crg1* or *mig1* promoter, resulting in the cbx^R or hyg^R carrying plasmids pHPcEAP, pCPcEAP, pCPmEAP. The construct was obtained by amplification of two fragments, cloned into the yeast vector pcrgPeb1$^{211–268}$ (ref. 3). A fragment encoding a 30 bp overhang for the *crg1* promoter and the first 739 amino acids of Kin1^{G96E} was amplified from the plasmid pCcrgKin1^{rigor} (ref. 7), using primers EB170 and EB171. A 437 bp fragment encoding the Yup1 PX domain (aa 4-148); an 30 bp overhang of *Tnos* terminator was amplified from the plasmid pSl-Yup-RFP-Hyg using primers EB172 and EB173. After linearization with *Sca*I and *BciV*I, the plasmid was integrated ectopically into AB33Kin3G_ChRab5a, AB33Mcs1G_3 and AB33G_3Dyn2_ChRab5a (ref. 8) resulting in strains AB33Kin3G_ChRab5a_cEAP, AB33Mcs1G_3_cEAP and AB33G_3Dyn2_ChRab5a_cEAP. pHPcEAP was derived from the plasmid pCPcEAP by replacement of the cbx^R with a hyg^R. It was linearized with *Ssp*I and *Psi*I and ectopically integrated into AB33GRab5a and SG200_GRab5a, resulting in strains AB33GRab5a_cEAP and

SG200_GRab5a_cEAP, respectively. pCPmEAP was derived from plasmid pCPcEAP by replacing the *crg* promoter with the *mig1* promoter[9], which was amplified from genomic DNA from strain SG200, using primers EB192 and EB193. The plasmid was linearized with *BsrG*I and ectopically integrated into the SG200_GRab5a, resulting in SG200_GRab5a_mEAP.

p∆Pit2. This plasmid was used to delete the effector gene *pit2* in *U. maydis*. To obtain p∆Pit2, plasmid pHrpl25G (ref. 10) was digested with *Sph*I, and geneticin resistance cassette (*G418*^{R}) from plasmid pMF1g (ref. 11); 1164 bp and 1161 bp fragments of upstream and downstream of *pit2* ORF were amplified by PCR using primers YH518, YH519, YH520, YH521, YH527 and YH528, introducing 30 bp overhang with the backbone of the vector. The plasmid p∆Pit2 was digested with *Eco*RI, *Psi*I and *Sna*BI and a 4440 bp fragment was integrated into the *pit2* locus of SG200GRab5a_cEAP, resulting in SG200GRab5a_cEAP_∆Pit2.

pCnPit2Ch and pHnPit2Ch. pCnPit2Ch was used to express *pit2-mCherry* under the *pit2* native promoter in the *pit2* deletion strain. This strategy was described previously [12]. 3121 bp upstream and the entire *pit2* ORF, excluding the stop codon, were amplified by PCR using YH539 and YH540. The fragment was used to replace the *cmu1* gene in either pCCmu1Ch or pHCmu1Ch. The plasmid pCnPit2Ch was digested with *Sca*I and the 9782 bp fragment was and integrated ectopically into SG200GRab5a_cEAP_∆Pit2, resulting in SG200GRab5a_cEAP_∆Pit2_nPit2Ch. pHnPit2Ch was digested with *Hpa*I and a 9995 bp fragment was integrated ectopically into the strains SG200 and $SG200G_3$, resulting in SG200nPit2Ch and $SG200G_3$_nPit2Ch, respectively.

p∆Rab5a. This plasmid was used to delete the *rab5a* gene in *U. maydis*. The backbone was derived from pCRII TOPO (Life Technologies Ltd, Paisley, UK) after digestion with *Xho*I and *Hind*III. A 1077 bp fragment upstream of the *rab5a* gene was amplified by PCR, thereby generating a *Sfi*I restriction site (using primers ODA148 and ODA149). A 1042 bp fragment downstream of the *rab5a* gene was amplified, generating a *SfiI* restriction site (primers ODA150 and ODA151). Both fragments and a *nat*^{R} cassette were ligated into plasmid pCRII TOPO. The plasmid was digested with *Hind*III and *Psp*XI and integrated into the *rab5a* locus of AB33, SG200 and SG200Pit2Ch, resulting in AB33∆Rab5a*,* SG200∆Rab5a and SG200Pit2Ch_∆Rab5a, respectively. Deletions of *rab5a* gene were confirmed by Southern Blot. EEs were visualised by ectopic integration of plasmid pYup1SG2 (ref. 6) into AB33∆Rab5a, resulting in AB33∆Rab5a_Yup1G strain.

pMcs1G$_3$. This plasmid contains 3 copies of *egfp* encoding region fused behind the *mcs1 gene* and *cbx*^{R}. The backbone was derived from pN_ERGFP (ref. 13), using *Nde*I and *Hind*III, and ligated with (i) a 582 bp of the *mcs1* ORF, excluding the stop codon (amplified with primers OWI114 and OWI120, thereby generating *Nde*I and *Nco*I restriction sites), (ii) a 525 bp fragment of the *mcs1* terminator (primers OWI116 and OWI117, generating sites for *Mul*I and *Hind*III), (iii) a 2991 bp fragment, containing the *hyg*^{R} from the plasmid pH4GFP (ref. 13), digested with *Bgl*II and *Mlu*I), and (iv) a fragment containing three copies of *egfp* and the *Tnos*, derived from plasmid pOG (ref. 14) by digestion with *Nco*I and *Bgl*II. pMcs1G$_3$ was digested with *BspH*I and integrated into the *mcs1* locus of AB33 resulting in AB33Mcs1G$_3$. Correct integration was confirmed by Southern Blot.

pKpp2G, pFuz7G, pKpp2G, pKpp6G, pCrk1G. The plasmids contain an appropriate gene under the *otef* promoter, fused to *egfp* and *cbx*^{R}. The constructs were obtained by amplification of a single fragment and cloned into the yeast vector poPXG (ref. 10) linearized with NgoM*IV*. A fragment encoding a 30 bp overhang for the *otef* promoter, *egfp* and the genes of Kpp2 (um03305), Kpp4 (um04258), Fuz7 (um01514), Gsk3β (um00560), Kpp6 (um02331) and Crk1 (um11410), respectively, was amplified from genomic DNA from strain 521 using sets of primers EB513-EB514, EB515-EB516, EB517-EB518, EB519-EB520, EB521-EB522 and EB523-EB524, respectively. After linearization with *Age*I or *Hpa*I the plasmids were integrated ectopically into AB33mChRab5a strain[4], resulting in AB33mChRab5a_Kpp2G, AB33mChRab5a_Kpp4G, AB33mChRab5a_Fuz7G, AB33mChRab5a_Gsk3β G, AB33mChRab5a_Kpp6G and AB33mChRab5a_Crk1G, respectively.

pCrk1G$_3$. The plasmid consists of a 1,065 bp fragment near the 3' end of the *crk1* gene followed by triple *egfp* and the *Tnos* terminator, the hygromycin resistance cassette, and a 1,060 bp fragment downstream of the *crk1* gene. The fragments were amplified with 30 bp overhangs from genomic DNA of the *U. maydis* strain 521 using sets of primers EB543-EB544 and EB545-EB546, respectively, and cloned with a 4,843 bp fragment encoding triple *egfp*, the *Tnos* terminator and the hygromycin cassette and a 5,536 bp fragment encoding an ampicillin resistance cassette, an *E. coli* replication origin, the yeast URA3 marker and *2 μ ori.* Both fragments were derived from the plasmid pHok1G$_3$ by digestion with *Psi*I and *BamH*I (a 4,843 bp fragment) and with *Bgl*I, *Xcm*I and *EcoN*I (a 5,536 bp fragment). The plasmid pCrk1G$_3$ was digested with *Apa*I and *BspH*I and homologously integrated into the *crk1* locus of strain AB33mChRab5a

(ref. 4) and SG200, resulting in AB33mChRab5a_Crk1G_3 and SG200Crk1G_3 respectively. Integration of triple *egfp* into the *crk1* locus was confirmed by PCR and Southern blotting.

p∆Crk1. This plasmid was used to delete the *crk1* gene in *U. maydis*. In this plasmid, the endogenous *crk1* gene is replaced by the *G418*^{R}. To obtain the plasmid, a 1,079 bp fragment, containing the *crk1* promoter (-1,001 bp), and a 1,079 bp fragment of genomic DNA, containing the downstream sequence of the *crk1* gene, were amplified by PCR using sets of primers EB580-EB581and EB582-EB583, respectively. Obtained fragments were cloned by *in vivo* recombination in the yeast *S. cerevisiae*, flanked by a 2,011 bp region encoding G418^R (amplified from the pMF1g [ref. 11] using primers EB475-EB476) into a 5,088-bp region of the pNEBcbx-yeast plasmid, which was digested by *EcoR*I and *Hind*III. The plasmid was digested with *EcoR*I and *Ssp*I and integrated into the *crk1* locus of SG200Cmu1Ch and SG200_*cmu*G, resulting in SG200Cmu1Ch_∆Crk1 and SG200*cmu*G_∆Crk1, respectively. Deletions of *crk1* gene were confirmed by Southern Blot.

Supplementary References

1 Raymond, C. K., Pownder, T. A. & Sexson, S. L. General method for plasmid construction using homologous recombination. *Biotechniques* **26**, 134-141 (1999).

2 Aichinger, C. *et al.* Identification of plant-regulated genes in *Ustilago maydis* by enhancer-trapping mutagenesis. *Mol Genet Genomics* **270**, 303-314, (2003).

3 Schuster, M. *et al.* Controlled and stochastic retention concentrates dynein at microtubule ends to keep endosomes on track. *EMBO J* **30**, 652-664, (2011).

4 Bielska, E. *et al.* Hook is an adapter that coordinates kinesin-3 and dynein cargo-attachment on early endosomes *J. Cell Biol.* **204**, 989-1007 (2014).

5 Straube, A., Hause, G., Fink, G. & Steinberg, G. Conventional kinesin mediates microtubule-microtubule interactions in vivo. *Mol Biol Cell* **17**, 907-916 (2006).

6 Wedlich-Söldner, R., Bölker, M., Kahmann, R. & Steinberg, G. A putative endosomal t-SNARE links exo- and endocytosis in the phytopathogenic fungus *Ustilago maydis*. *EMBO J* **19**, 1974-1986 (2000).

7 Schuster, M. *et al.* Myosin-5, kinesin-1 and myosin-17 cooperate in secretion of fungal chitin synthase. *EMBO J* **31**, 214-227 (2012).

8 Schuster, M., Lipowsky, R., Assmann, M. A., Lenz, P. & Steinberg, G. Transient binding of dynein controls bidirectional long-range motility of early endosomes. *Proc Natl Acad Sci U S A* **108**, 3618-3623 (2011).

9 Basse, C. W., Stumpferl, S. & Kahmann, R. Characterization of a *Ustilago maydis* gene specifically induced during the biotrophic phase: evidence for negative as well as positive regulation. *Mol Cell Biol* **20**, 329-339 (2000).

10 Higuchi, Y., Ashwin, P., Roger, Y. & Steinberg, G. Early endosome motility spatially organizes polysome distribution. *J Cell Biol* **204**, 343-357 (2014).

11 Baumann, S., Pohlmann, T., Jungbluth, M., Brachmann, A. & Feldbrügge, M. Kinesin-3 and dynein mediate microtubule-dependent co-transport of mRNPs and endosomes. *J Cell Sci* **125**, 2740-2752 (2012).

12 Doehlemann, G., Reissmann, S., Assmann, D., Fleckenstein, M. & Kahmann, R. Two linked genes encoding a secreted effector and a membrane protein are essential for *Ustilago maydis*-induced tumour formation. *Mol Microbiol* **81**, 751-766 (2011).

13 Straube, A., Weber, I. & Steinberg, G. A novel mechanism of nuclear envelope break-down in a fungus: nuclear migration strips off the envelope. *EMBO J* **24**, 1674-1685 (2005).

14 Lenz, J. H., Schuchardt, I., Straube, A. & Steinberg, G. A dynein loading zone for retrograde endosome motility at microtubule plus-ends. *EMBO J* **25**, 2275-2286 (2006).

15 Doehlemann, G. *et al.* Pep1, a secreted effector protein of *Ustilago maydis*, is required for successful invasion of plant cells. *PLoS Pathog* 5, e1000290 (2009).

16 Djamei, A. *et al.* Metabolic priming by a secreted fungal effector. *Nature* **478**, 395-398 (2011).
